# Overall avidity declines in TCR repertoires during latent CMV but not EBV infection

**DOI:** 10.3389/fimmu.2023.1293090

**Published:** 2023-11-20

**Authors:** Barbara Couturaud, Bastien Doix, Laura Carretero-Iglesia, Mathilde Allard, Sylvain Pradervand, Michael Hebeisen, Nathalie Rufer

**Affiliations:** ^1^ Department of Oncology, Lausanne University Hospital and University of Lausanne, Epalinges, Switzerland; ^2^ Lausanne Genomic Technologies Facility (LGTF), University of Lausanne, Lausanne, Switzerland

**Keywords:** healthy donors, longitudinal study, latent herpesvirus infection, CD8 T cells, TCR clonotype, persistence, TCR off-rates, LILRB1

## Abstract

**Introduction:**

The avidity of the T-cell receptor (TCR) for antigenic peptides presented by the MHC (pMHC) on cells is an essential parameter for efficient T cell-mediated immunity. Yet, whether the TCR-ligand avidity can drive the clonal evolution of virus antigen-specific CD8 T cells, and how this process is determined in latent Cytomegalovirus (CMV)- against Epstein-Barr virus (EBV)-mediated infection remains largely unknown.

**Methods:**

To address these issues, we quantified monomeric TCR-pMHC dissociation rates on CMV- and EBV-specific individual TCRαβ clonotypes and polyclonal CD8 T cell populations in healthy donors over a follow-up time of 15-18 years. The parameters involved during the long-term persistence of virus-specific T cell clonotypes were further evaluated by gene expression profiling, phenotype and functional analyses.

**Results:**

Within CMV/pp65-specific T cell repertoires, a progressive contraction of clonotypes with high TCR-pMHC avidity and low CD8 binding dependency was observed, leading to an overall avidity decline during long-term antigen exposure. We identified a unique transcriptional signature preferentially expressed by high-avidity CMV/pp65-specific T cell clonotypes, including the inhibitory receptor LILRB1. Interestingly, T cell clonotypes of high-avidity showed higher LILRB1 expression than the low-avidity ones and LILRB1 blockade moderately increased T cell proliferation. Similar findings were made for CD8 T cell repertoires specific for the CMV/IE-1 epitope. There was a gradual in vivo loss of high-avidity T cells with time for both CMV specificities, corresponding to virus-specific CD8 T cells expressing enhanced LILRB1 levels. In sharp contrast, the EBV/BMFL1-specific T cell clonal composition and distribution, once established, displayed an exceptional stability, unrelated to TCR-pMHC binding avidity or LILRB1 expression.

**Conclusions:**

These findings reveal an overall long-term avidity decline of CMV- but not EBV-specific T cell clonal repertoires, highlighting the differing role played by TCR-ligand avidity over the course of these two latent herpesvirus infections. Our data further suggest that the inhibitor receptor LILRB1 potentially restricts the clonal expansion of high-avidity CMV-specific T cell clonotypes during latent infection. We propose that the mechanisms regulating the long-term outcome of CMV- and EBV-specific memory CD8 T cell clonotypes in humans are distinct.

## Introduction

Primary Cytomegalovirus (CMV) and Epstein-Barr virus (EBV) infection is associated with a robust T cell response, followed by the establishment of a lifelong latency, while the T cell response persists long-term. In healthy individuals, herpesviruses are generally well controlled, through a fine balance between viral determinants and host immune surveillance. Yet, CMV and EBV cause considerable morbidity and mortality, particularly in immunocompromised individuals (1, 2). The maintenance of memory CD8 T cell responses during CMV and EBV latent infection have been shown to differ in terms of frequency, phenotype and function. Whereas the prevalence of EBV-specific memory CD8 T cells generally remains stable over time in healthy carriers (3, 4), CMV-specific T cells with certain specificities slowly accumulate as infection advances into latency until reaching high and stably maintained frequencies, in a process named “memory inflation” (5–8). Moreover, CD8 T cells specific for EBV mostly display a less differentiated memory phenotype than the CMV-specific T cell pool, that typically harbor a mature phenotype (9, 10) and share functional features (i.e. cytokine production and constitutive cytolytic activity) common among acute effector cells (9, 11–14). It is becoming increasingly clear that repetitive exposure to antigen is a key determinant of memory T cell inflation/expansion during CMV latent infection. Indeed, CMV is characterized by intermittent reactivation from latency, representing a low-level persistent infection, which subsequently impacts on the virus-specific CD8 T cell response (15). Conversely, EBV reactivation during latency is thought to occur only occasionally, with virus-specific T cells showing no sign of expansion/inflation comparable to CMV infection (16).

Extensive research has been undertaken to determine which main drivers are underlying the selection and long-term maintenance of the large populations of CMV-specific memory CD8 T cells (8, 17). Competition for antigen at the level of the antigen presenting cell (18), as well as between virus-specific T cells (19), have been shown to favor clonal dominance during memory inflation induced by murine CMV (MCMV) infection. Other studies have implicated a dominant role for the immunoproteasome (20) and for the viral gene expression context (21) in defining T cell immunodominance against MCMV. More recently, latent MCMV infection of lymphatic endothelial cells, where sporadic reactivation events occur, has been shown to be sufficient to drive CD8 T cell memory inflation (22). Along the same lines, Welten and colleagues identified a subset of Tcf1+ MCMV-specific CD8 T cells which is able to maintain the peripheral cell pool of inflationary T cells (23). Another key observation is that memory CMV-specific CD8 T cell repertoires are often described as highly oligoclonal with an initial skewing following primary infection, resulting in a limited clonal diversity through the latency infection phase (5, 10, 24–26). Hence, it has been proposed that the strength of TCR-pMHC interactions (defined thereafter as TCR avidity) represents a major determinant of the TCR repertoire selection and dominance of human CMV-specific CD8 T cell clonotypes (10, 24–27). Investigations of TCR repertoire evolution during aging have suggested an accumulation of CMV-specific CD8 T cells of lower avidity or functional potential in elderly individuals (28–31). However, most of these studies only rely on cross-sectional analyses of different age groups and/or on short timespan evaluation. In a recent elegant study, high-avidity T cell clones were found to decline during the chronic phase of MCMV infection, leading to a switch in dominance towards low-avidity T cells (32). Nonetheless, there is a continued need to improve our knowledge about the intrinsic role of TCR-ligand avidity on T cell clonotype responses during the long-term course of human latent herpesvirus infection.

We had the unique opportunity to study the parameters driving TCRαβ clonal evolution between CMV and EBV chronic infection from six HLA-A2-positive healthy individuals in a longitudinal study, during an observational period of up to 18 years. Specifically, we assessed the structural TCR-pMHC avidity by monomeric TCR-ligand off-rate measurements (33, 34) combined to gene expression profiling and phenotypic and functional characterization of the clonal repertoire in CMV-specific (HLA-A2/pp65_495-503_ and HLA-A2/IE-1_316-324_) versus EBV-specific (HLA-A2/BMFL1_280-288_) CD8 T cells over time. Our findings reveal a critical role played by TCR avidity-driven repertoire evolution in the long-term outcome (i.e. 15 to 18 years) of CMV-specific but not of EBV-specific CD8 T cell responses in healthy individuals. LILRB1 inhibitor checkpoint was also preferentially expressed in high-avidity CMV-specific T cell clonotypes and may potentially limit their life expectancy during persistent infection.

## Materials and methods

### Ethics statement

This study protocol was reviewed and approved according to the relevant regulatory standards from the Ethics Committee for Clinical Research of the University of Lausanne (Lausanne, Switzerland) in accordance with the Declaration of Helsinki. All healthy donors have provided written informed consent for the use of blood samples in medical research.

### Healthy donors

Leukapheresis were collected at Interregional Blood Transfusion SRC from six HLA-A2-positive healthy individuals latently infected with the CMV and/or EBV viruses (BCL1, BCL2, BCL4, BCL6, BCL7 and BCL9) in 2002 (i.e. time-point T_n_) as described previously (10). All donors had previously completed the Swiss National Medical questionary to verify that they fulfilled the criteria of apheresis donation. Blood samples from the same individuals were collected 15 to 18 years later (i.e. time-point T_n+15y_ and T_n+18y_). No clinical data were available on their acute infection in the past, but all individuals remained in excellent health during this long-term follow-up. Peripheral blood mononuclear cells (PBMCs) centrifuged in Ficoll-Hypaque (Pharmacia) were cryopreserved in 10% DMSO and stored in liquid nitrogen until further use.

### TCRαβ clonotype repertoire sequencing

Thawed PBMCs were positively enriched using anti-CD8-coated magnetic microbeads (Miltenyi Biotec), stained in PBS, 0.2% BSA, and 5 mM EDTA with PE-labeled HLA-A*0201 multimers loaded with native CMV/pp65_495-503_ (NLVPMVATV) or EBV/BMFL1_280-288_ (GLCTLVAML) peptide (Peptide and Tetramer Core Facility, CHUV/UNIL/LICR, Lausanne, Switzerland) at 4°C for 45 minutes, followed by cell surface marker APC-A750 anti-CD8 (Beckman Coulter) at 4°C for 30 minutes. Virus-specific CD8 T cells (CD8^+^multimer^+^) were sorted on a FACS Aria (BD Biosciences) flow cytometer as 300-1000 cells, before further cloning by limiting dilution and expansion in RPMI 1640 medium (Gibco) supplemented with 8% human serum, 150 U/ml human recombinant IL-2 (a gift from GlaxoSmithKline), 1 μg/ml PHA (Sodiag), and 1×10^6^/ml 30 Gy-irradiated allogeneic PBMCs as feeder cells. T cell clones (2x10^4^ cells) were directly processed through direct cell lysis and cDNA synthesis. cDNA sample was then subjected to individual PCR using a set of previously validated forward primers specific for the different known *TRBV* or *TRAV* gene subfamilies and two reverse primers specific for the corresponding *C-beta* or *C-alpha* gene segments (10, 35). PCR products of interest were sequenced from the reverse primer (Fasteris SA). Clonotypes were defined as T cell clones sharing the same *TRBV-CDR3* and *TRAV-CDR3* amino acid sequences. TCR sequences were analyzed using SnapGene (v.4.1.9 GSL Biotech) and described according to the IMmunoGeneTics (IMGT) nomenclature (36).

### Direct *ex vivo* TRBV family and clonotype repertoire analyses

CD8-enriched T cells from PBMCs were initially stained at 4°C for 45 minutes with CMV/pp65-specific or EBV/BMFL1-specific multimers, followed by cell surface marker APC-A750, FITC or APC anti-CD8 (Beckman Coulter) and antibodies against the different identified TRBV families (**Supplementary Table 1**) at 4°C for 30 minutes. Samples were acquired on a LCRII cytometer (BD Biosciences) and analyzed using FlowJo 10.4.2 software (v.10.4.2, Tree Star). The frequency of CMV/pp65-specific TRBV clonotypes was further assessed for each *ex vivo* TRBV family identified by flow cytometry from donors BCL4 and BCL6. Briefly, virus-specific CD8 T cells, which stained positive for a given TRBV family were sorted, *in vitro* cloned and *TRBV-CDR3* sequenced as described above.

### NTAmer-based dissociation kinetics

NTAmers were synthetized by the Peptide and Tetramer Core Facility, Ludwig Cancer Research, UNIL CHUV (Lausanne, Switzerland) as described previously (37). Dually labeled NTAmers are composed of streptavidin-phycoerythrin (SA-PE) complexed with biotinylated peptides and non-covalently bound to His-tagged HLA-A*0201 monomers containing Cy5-labeled β2m (37), and were used for dissociation kinetic measurements as described previously (33, 34). Individual virus-specific CD8 T cell clones or bulk virus-specific CD8 T cell populations expanded following short-term *in vitro* stimulation with PHA and irradiated feeder cells were stained for 45 minutes at 4°C in PBS, 0.2% BSA and 5 mM EDTA with virus-specific NTAmers, in which the HLA-A*0201 molecules were either loaded with native EBV/BMFL1_280-288_ (GLCTLVAML), CMV/pp65_495-503_ (NLVPMVATV) or CMV/IE-1_316-324_ (VLEETSVML) peptides. NTAmers prepared with CD8 binding-deficient HLA-A*0201 monomers (i.e. CD8-null NTA) bearing the D227K/T228A mutations in the HLA α3 domain (33) were used when indicated. To assess the differential dissociation kinetics relative to LILRB1 expression, CD8 T cells were first stained with SB600 anti-CD85j antibody (Invitrogen) for 20 minutes at room temperature before NTAmer staining. NTAmer staining was analyzed at 4°C on a SORP-LSR II cytometer (BD Biosciences). Following 30 seconds of baseline acquisition, imidazole (100 mM) was added allowing for the rapid dissociation of the SA-PE-NTA_4_ scaffold and monomeric Cy5 fluorescence was measured during the following 5 to 10 minutes. Data were analyzed using the kinetic module of FlowJo software (v.9.7.6, Tree Star) and modeled (1-phase exponential decay) using Prism software (v.9, GraphPad).

### *Ex vivo* global transcriptome profiling by RNA sequencing analysis

CD8-enriched from PBMCs were stained with CMV/pp65-specific multimers followed by cell surface marker APC-A750, FITC or APC anti-CD8 (Beckman Coulter) and antibodies against the different identified TRBV families (**Supplementary Table 1**). CMV/pp65/TRBV-specific CD8 T cells were then sorted on a FACSAria (BD Biosciences) flow cytometer in RNAlater Stabilization Solution (Invitrogen). Total RNA from the *ex vivo* sorted cells (between 300 and 8000 cells) was extracted using the RNeasy Micro Kit (Qiagen) according to the manufacturer’s protocol. RNA-sequencing libraries were prepared using the Clontech SMART Seq v4 Ultra Low RNA kit (Clontech Laboratories, Inc.) and sequencing was performed on the Illumina HiSeq 2500. Library preparation, sequencing and analyses were performed by the Lausanne Genomic Technologies Facility (UNIL, Lausanne, Switzerland) as described in detail in the Supplemental Data section. Gene expression data have been deposited in the NCBI Gene Expression Omnibus (GSE246111).

### *Ex vivo* phenotype expression analysis

CD8-enriched T cells (Miltenyi Biotec) were isolated from thawed healthy donors PBMCs and stained in PBS, 0.2% BSA and 5 mM EDTA with AF700 anti-CD8 (Biolegend), APC-efluor780 anti-CD28 (eBioscience), ECD anti-CD45RA (Beckman Coulter), APC or SB600 anti-CD85j (LILRB1, Invitrogen) and BV421 anti-CD57 (BD Biosciences) for 30 minutes at room temperature. When required CD8^pos^ T cells were further stained with appropriate TRBV antibodies to discriminate high- versus low-avidity CMV/pp65-specific TCRαβ clonotypes (**Supplementary Table 1**). Cells were stained with PE-labeled HLA-A*0201 multimers loaded with native CMV/pp65_495-503_, CMV/IE-1_316-324_ or EBV/BMFL1_280-288_ peptide for 45 minutes at 4°C. Vivid Aqua (Invitrogen) staining (20 minutes at 4°C in PBS) was used to discriminate live/dead cells. LSRII (BD Biosciences) and Cytoflex (Beckman Coulter) flow cytometers were used to acquire the data. The percentage of positive cells or the level of expression of each marker (geometric mean fluorescence intensity [gMFI]) were analyzed using FlowJo software (v.10.4.2, Tree Star). For the dimensional reduction analyses, samples at each time-point were downsized using the downsample V3 plugin (FlowJo, v.10.4.2) before concatenation and analysis with both the t-SNE built-in module and the FlowSOM plugin (38). FlowSOM plugin was run with the following settings: 5 metaclusters and a SOM grid of 8x8 with a seed of 3. The number of CMV/pp65- or CMV/IE-specific CD8 T cells from BCL6 shown at T_n_ and T_n+18y_ was adjusted by downsampling to be proportional to the multimer fractions found at each time-point.

### *In vitro* LILRB1 blocking experiments

30-Gy-irradiated HLA-A*0201-positive PBMCs were pulsed 1 hour at 37°C with the native CMV/pp65_495-503_ peptide (10^-8^M), washed, and incubated with CFSE-labelled (Invitrogen) CMV/pp65-specific CD8 T cell clones of high or low avidity at an E/T ratio of 1:1 in RPMI 1640 medium (Gibco) supplemented with 8% human serum and 50 U/ml human recombinant IL-2 (GlaxoSmithKline) in the presence of monoclonal IgG_2B_ mouse anti-human LILRB1 (CD85j, ILT2) blocking antibody or an IgG_2B_ isotype control used at 5 μg/ml (R&D Systems). After 3 days, cells were washed, stained in PBS with Near-IR Vivid (Invitrogen) at 4°C for 30 minutes before acquisition on a LSRII (BD Biosciences) cytometer. The percentage of divided cells, as well as the expansion and replication indexes were determined using the proliferation analysis tools available in FlowJo (v.10.4.2, Tree Star).

### Statistical analyses

Data were analyzed using the GraphPad Prism software (v.9 or v.10.0.1, www.graphpad.com), Statistical tests were used as indicated throughout the manuscript.

## Results

### TCR clonotype dominance for CMV/pp65 but not EBV/BMFL1 varies over time

**Figure 1:**
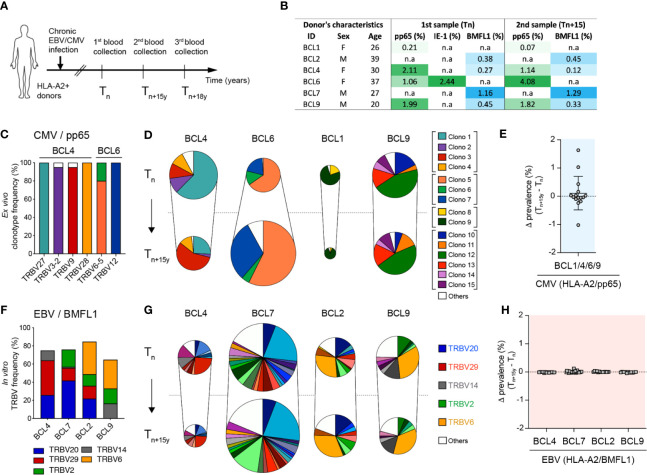
Evolution of CMV/pp65- and EBV/BMFL1-specific CD8 TCRαβ clonotypes in longitudinal analyses. **(A)** Schematic representation of donor sampling at early (T_n_) and late (T_n+15y_, T_n+18y_) time-points during latent CMV and/or EBV infection. **(B)** Donor characteristics and frequencies of CMV (HLA-A*0201/pp65_495-503_)- and/or EBV (HLA-A*0201/BMFL1_280-288_)-specific T cells within total CD8 T populations over time. Of note, CMV (HLA*0201/IE-1_316-324_) specificity was only detectable in BCL6 and is shown at T_n_. **(C)** Frequencies of color-coded CMV/pp65-specific clonotypes per TRBV family for BCL4 and BCL6. Specific *TRBV-CDR3* clonotypes were determined following *ex vivo* FACS-sorting of TRBV-positive CMV/pp65-specific T cells, *in vitro* single-cell cloning and *TRBV-CDR3* sequencing. **(D)** Quantification of co-dominant CMV/pp65-specific TCRαβ clonotypes at T_n_ and T_n+15y_, adjusted to the CMV/pp65-specific population size (multimer^+^CD8^+^ T cells). Results are presented as percentages of color-coded CMV/pp65-specific T cell clonotypes (n = 1108 *in vitro* generated clones). **(E)** Delta-prevalence or variation in clonotype prevalence from **(D)** (i.e. subtraction T_n+15y_ - T_n_ of each individual TCRαβ clonotype frequency within total CD8 T cells) for the CMV/pp65 specificity. **(F)** Cumulative *in vitro* single cell cloning-based frequencies of preferential TRBV family usage within EBV/BMFL1-specific clonotypes at T_n_. **(G)** Quantification of co-dominant EBV/BMFL1-specific TCRαβ clonotypes at T_n_ and T_n+15y_, adjusted to the EBV/BMFL1-specific population size (multimer^+^CD8^+^ T cells). Results are presented as percentages of EBV/BMFL-1-specific T cell clonotypes classified according to their color-coded TRBV family (n = 1114 *in vitro* generated clones). **(D, G)** Unique clonotypes are defined as “others” and depicted in white. For each donor, *in vitro* single-cell cloning of T_n_ and T_n+15y_ samples was performed alongside. **(H)** Variation in clonotype prevalence from **(G)** (i.e. subtraction T_n+15y_ - T_n_ of each individual TCRαβ clonotype frequency within total CD8 T cells) for the EBV/BMFL1 specificity. Data are presented according to the indicated healthy donors.

Previous reports have shown that CD8 T cell clonal repertoires in response to chronic CMV or EBV infection are highly stable over time (10, 39–42). Yet, the maximal timespan analyzed in those studies was of five years, whereas CMV and EBV immune responses persist for decades. To evaluate how lifelong persistent CMV and EBV infections might impact on the TCRαβ clonal evolution, we performed an in-depth clonotype repertoire analysis on virus-specific CD8 T cells from six healthy donors latently infected with CMV and/or EBV, during an observational period of 15 to 18 years (**Figure 1A**). The proportion of CMV/pp65_495-503_-, CMV/immediate-early (IE)-1_316-324_- and EBV/BMFL1_280-288_-specific populations within total CD8 T cells over time was monitored by *ex vivo* fluorescent multimer staining (**Figure 1B**; **Supplementary Figure 1A**). No consistent pattern of frequency evolution was found among CMV/pp65-specific T cells, as that of donor BCL6 increased with time (from 1.06% to 4.08%), while the other three individuals showed reduced frequencies across the two time-points with some donor-related variations (between 0.14% to 0.97%). CMV/IE-1-specific T cells were only detectable in donor BCL6, in agreement with its previously described low prevalence in CMV-seropositive HLA-A*0201-expressing individuals (43). In contrast, EBV/BMFL1-specific T cell populations were globally more stable during the period of 15 years, with a maximum frequency variation of 0.15% (**Figure 1B**; **Supplementary Figure 1A**).

Within CMV/pp65 and EBV-BMFL1 specificities, we assessed the evolution of the TCRαβ clonal repertoires over time on *ex vivo* CD8 T cells or large panels of single T cell clones derived by *in vitro* limiting dilution cultures. The clonal composition was analyzed based on *TRBV-CDR3* and *TRAV-CDR3* gene sequences (10, 35). To validate the TCRαβ repertoires obtained from *in vitro*-generated virus-specific T cell clones, we used a panel of TCRβ chain-targeted (i.e. TRBV) antibodies combined with epitope-specific multimers for the *ex vivo* staining of CMV/pp65- or EBV/BMFL1-specific CD8 T cells at the TRBV level (**Supplementary Figure 1B**). Robust correlations were obtained for both epitope specificities with similar frequencies of co-dominant TRBV families as determined by the two independent approaches (**Supplementary Figure 1C**) and in agreement with previous studies (10, 35, 44). In addition, TRBV-positive CMV/pp65-specific CD8 T cells were directly FACS-sorted, *in vitro* cloned and sequenced for *TRBV-CDR3*, revealing that over 95% of all TRBV family-recovered clones bore the corresponding *TRBV-CDR3* clonotype (**Figure 1C**). These data indicate that *ex vivo* TRBV family staining is a powerful indicator of CMV/pp65-specific T cell clonotype frequencies, as these are mostly composed of few well-defined co-dominant TCRαβ clonotypes bearing distinct TRBV families (**Supplementary Table 2**).

All CMV/pp65-specific clonotypes identified at the early time-point were also found 15 years later (**Figure 1D**), revealing a notable long-term persistence of the highly restricted clonal repertoires. However, when adjusted to the population size, fluctuations were still observed with certain clonotypes showing increased or decreased prevalence over time (**Figures 1D, E**). These changes in clonal dominance were also found when analyzing CMV/pp65-specific populations at the direct *ex vivo* TRBV-chain family level using a panel of TRBV-targeted antibodies (**Supplementary Figure 1D**). This was more evident for donors BCL4, BCL6 and BCL1 than for BCL9, presenting a relative stable TCR clonotype repertoire over the studied period. Despite presenting a diverse clonotype composition with 10 to 22 codominant clonotypes per donor, EBV/BMFL1-specific T cell repertoires were highly biased in their *TRBV* and *TRAV* family usage (**Figure 1F**; **Supplementary Table 3**), as previously reported (10, 25, 45–47). Contrasting to CMV/pp65-specific CD8 T cell repertoires, the EBV/BMFL1 clonal ones were highly stable over time, with only minor variations in the adjusted TCR clonotype prevalence between time-points and per healthy individual (**Figures 1G, H**).

### TCR-pMHC dissociation rate is a stable intrinsic biomarker at the TCR clonotype level

**Figure 2:**
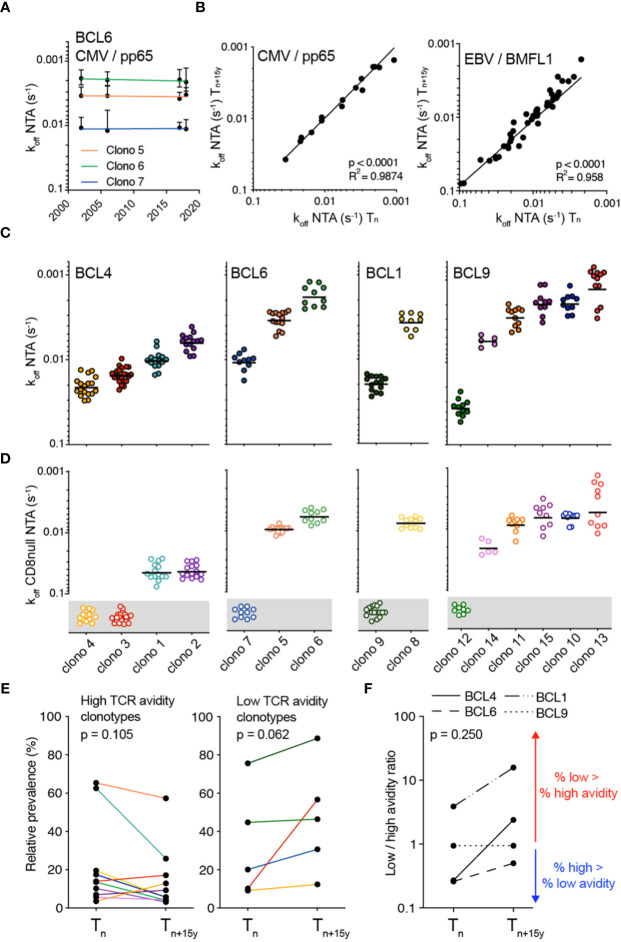
Monomeric TCR-pMHC dissociation rates of CMV/pp65-specific TCRαβ clonotypes in longitudinal analyses. **(A)** TCR-pMHC dissociation rates (k_off_) by wild-type NTAmers of each color-coded CMV-specific TCRαβ clonotypes from donor BCL6 at the indicated time-points. **(B)** Correlations of NTAmer-based TCR off-rates (k_off_) between T_n_ and T_n+15y_ obtained from identical TCRαβ clonotypes for CMV/pp65 (left panel) or EBV/BMFL1 (right panel) specificity. Coefficient R^2^ and p-values from simple linear regression analyses are indicated. **(C, D)** TCR-pMHC off-rates (k_off_) by wild-type NTAmers (**C**; NTA) or mutated NTAmers (**D**; CD8null NTA) on a representative selection of T cell clones of each identified TCRαβ clonotype and donor. CD8null NTA non-binder clones are represented in the grey boxes and are defined as CD8 binding-dependent clonotypes. The middle line represents the mean. **(E)** Relative prevalence of each CMV/pp65-specific TCRαβ clonotype at T_n_ and T_n+15y_ categorized as high TCR binding avidity/CD8 binding-independent (left panel) or as low TCR binding avidity/CD8 binding-dependent (right panel). Clonotypes are color-coded as in **Figure 1D**. **(F)** Ratio of low (CD8 binding-dependent)/high (CD8 binding-independent) TCR binding avidities within the overall CMV/pp65-specific CD8 T cell repertoires at T_n_ and T_n+15y_ for each indicated donor. Values >1 (red arrow) indicate an enrichment of low over high avidity CMV/pp65-specific T cell clonotypes. Values <1 (blue arrow) indicate an enrichment of high over low avidity T cell clonotypes. **(E, F)** p-values by Wilcoxon non-parametric paired test (p > 0.05, not significant).

We and others reported that the TCR-ligand off-rate (i.e. k_off_) is a reliable parameter, independent of the activation state of the T cell (34, 48). Hence, we further explored its robustness by comparing k_off_ from CMV/pp65-specific CD8 T cell clones expressing the same TCRαβ clonotype, but isolated over 15 to 18 years, using reversible 2-color NTAmers (i.e. NTA) (33). Each specific clonotype showed a remarkable stability of TCR-pMHC off-rate measurements over time (**Figure 2A**; **Supplementary Figure 2A**), translating into strong positive correlation between k_off_ rates obtained at T_n_ and T_n+15y_ from the four studied individuals (**Figure 2B**, left panel). Similar observations were made for the TCRαβ clonotypes specific for EBV/BMFL1 (**Figure 2B**, right panel), or when using mutated NTAmers, that were deficient for CD8 binding to pMHC (i.e. CD8-null NTA) (**Supplementary Figures 2B–D**). This indicates that for a particular CMV/pp65- or EBV/BMFL1-specific TCRαβ clonotype, the TCR-pMHC off-rate (i.e. NTA and CD8-null NTA) represents a stable intrinsic readout, unrelated to the time-point of blood sampling.

### Preferential long-term enrichment of low avidity CMV/pp65-specific T cell clonotypes

We next sought to determine whether the changing patterns of dominance observed between CMV/pp65-specific T cell clonotypes throughout the course of latent infection could be related to disparities in TCR-pMHC binding avidities. For each donor-specific clonal repertoire, NTA- and CD8-null NTA-based dissociation measurements allowed the identification of two distinct subgroups (**Figures 2C, D**). The first one was composed of CMV/pp65-specific T cell clonotypes of high binding avidity (i.e. slow off-rates/high k_off_ and low CD8 binding dependency as shown by their capacity to bind CD8-null NTAmers), as depicted by clono 1 and 2 (BCL4), clono 5 and 6 (BCL6) and clono 8 (BCL1). Conversely, the second subgroup was characterized by TCRs of low binding avidity (i.e. fast off-rates/low k_off_ and high CD8 binding dependency), such as clono 3 and 4 (BCL4), clono 7 (BCL6) and clono 9 (BCL1). Moreover, the relative prevalence of most high avidity clonotypes showed a decline over time, contrasting to the increase in relative frequency of the low avidity ones (**Figure 2E**). At the level of overall donor-specific repertoires, this led to a rise from T_n_ to T_n+15y_ in the ratios of low/high clonotype avidity (**Figure 2F**). The only exception was BCL9 with no major changes in the TCR repertoire avidity ratio over time, yet clono 12 with the lowest TCR binding avidity (**Figures 2C, D**) was the most prevalent clonotype, representing nearly 50% of the CMV/pp65-specific repertoire (**Figure 1D**). Due to the limited number of available donors (n = 4) with such long timespans (i.e. 15 to 18 years), data do not reach levels of statistically significant differences, but suggest a preferential long-term enrichment of low avidity clonotypes (i.e. fast TCR-pMHC dissociation rates and greater dependency on CD8 coreceptor binding) within CMV/pp65-specific CD8 T cell repertoires.

### TCR binding avidity does not shape the clonotype evolution for EBV/BMFL1

**Figure 3:**
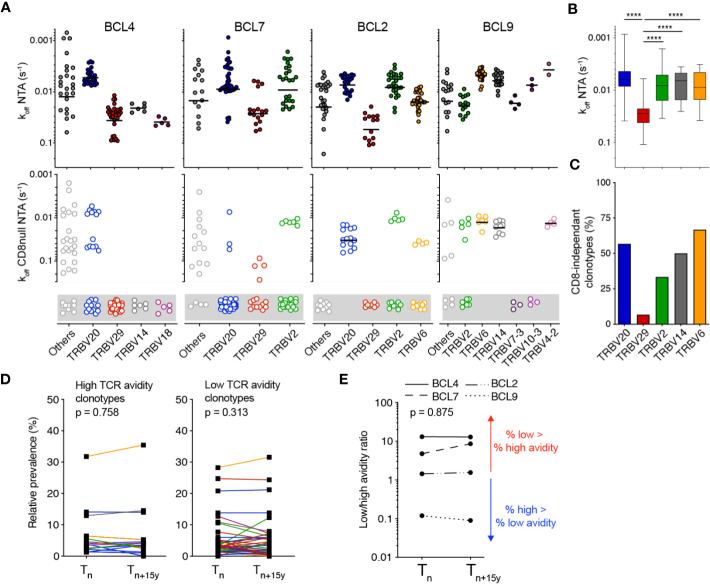
Monomeric TCR-pMHC dissociation rates of EBV/BMFL1-specific TCRαβ clonotypes in longitudinal analyses. **(A)** TCR-pMHC off-rates (k_off_) by wild-type NTAmers (NTA; top panels) or mutated NTAmers (CD8null NTA, bottom panels) on a representative selection of EBV/BMFL-1-specific T cell clones of each identified TCRαβ clonotype and donor, classified according to the TRBV family usage. CD8null NTA non-binder clones are represented in the gray boxes and are defined as CD8 binding-dependent clonotypes. The middle line represents the mean. **(B)** Compiled TCR-pMHC off-rates (k_off_ wild-type NTA) data from [**(A)**, top panels], classified according to their preferential TRBV family usage and depicted as box (25^th^ and 75^th^ percentiles) and whisker (min to max) plots with the middle line indicating the median. p-values by Kruskal-Wallis test; ^****^p < 0.0001. **(C)** Compiled TCR-pMHC off-rates (k_off_ CD8null NTA) data from [**(A)**, bottom panels] showing the proportion of CD8-independent TCRαβ clonotypes, classified according to their color-coded TRBV family. **(D)** Relative prevalence of each EBV/BMFL1-specific TCRαβ clonotype at T_n_ and T_n+15y_ categorized as high TCR binding avidity/CD8 binding-independent (left panel) or as low TCR binding avidity/CD8 binding-dependent (right panel). Clonotypes are color-coded as in **Figure 1G**. **(E)** Ratio of low (CD8 binding-dependent)/high (CD8 binding-independent) TCR binding avidities within the overall EBV/BMFL1-specific CD8 T cell repertoires at T_n_ and T_n+15y_ for each indicated donor. Values >1 (red arrow) indicate an enrichment of low over high avidity EBV/BMFL1-specific T cell clonotypes. Values <1 (blue arrow) indicate an enrichment of high over low avidity T cell clonotypes. **(D, E)** p-values by Wilcoxon non-parametric paired test (p > 0.05, not significant).

We next investigated the impact of TCR-binding avidity on individually identified TCRαβ clonotypes for EBV/BMFL1 using WT (i.e. NTA) and CD8 binding-deficient (i.e. CD8-null NTA) NTAmers. Similar to the CMV/pp65 clonal repertoires (**Figures 2C, D**), the TCRαβ clonotypes covered a broad spectrum of TCR binding avidity (i.e. k_off_ NTA) and CD8 binding capacity (**Figure 3A**; **Supplementary Figures 3A, B**). Moreover, TCR binding avidity and CD8 binding-dependency were closely related to distinct TRBV family usage (**Figures 3B, C**), but to a weaker extent to TRAV (**Supplementary Figure 3C**). Specifically, TRBV29 clonotypes displayed significantly faster TCR off-rates (i.e. lower k_off_) and were largely CD8 binding-dependent compared to TRBV20 clonotypes. Both high and low avidity EBV/BMFL1-specific TCR clonotypes displayed a great stability in terms of relative frequency during the period of 15 years (**Figure 3D**). As a consequence, the overall ratios of low/high TCR clonotype avidity, for each donor, remained stable over time (**Figure 3E**). In conclusion, differing from the CMV/pp65 model, TCR-pMHC binding avidity does not appear to shape the clonotype evolution of EBV/BMFL1-specific CD8 T cells during the course of latent infection.

### Progressive long-term avidity declines of CMV/pp65- but not EBV/BMFL1-specific CD8 T cell repertoires

**Figure 4:**
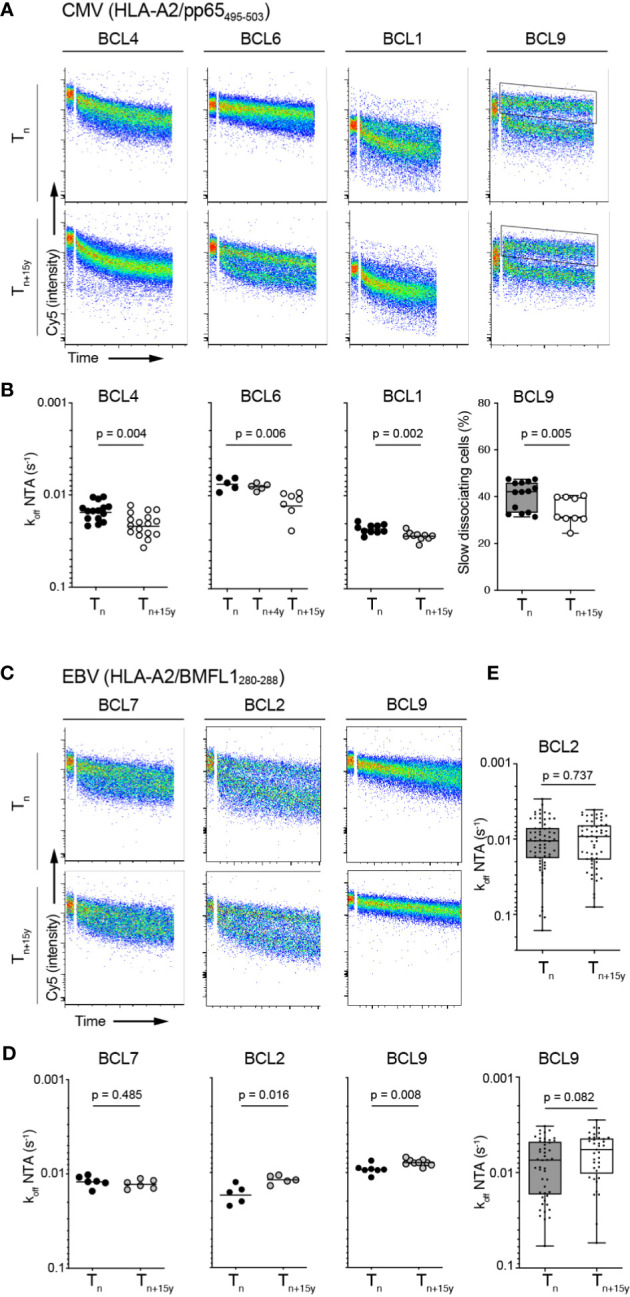
Overall TCR-pMHC dissociation rates on bulk CMV/pp65- and EBV/BMFL1-specific CD8 T cell populations in longitudinal analyses. **(A)** Representative FACS-based wild-type NTAmer dissociation curves obtained from short-term *ex vivo* expanded CMV/pp65-specific bulk T cell populations at T_n_ and T_n+15y_. Insets show the gating region used to estimate the proportion of T cells of slower off-rates in donor BCL9. **(B)** Data are representative of pooled dissociation rate (k_off_) values from 1 to 3 independent experiments. The mean value is indicated. For BCL9, percentage of slow dissociating CMV/pp65-specific bulk cells at T_n_ and T_n+15y_ based on the quantification of slow FACS-gated dissociation curves [see insets in **(A)**] is depicted. p-values by Mann-Whitney test (BCL4, BCL1, BCL9) and Kruskal-Wallis test (α = 0.05) (BCL6). **(C)** Representative FACS-based wild-type NTAmer dissociation curves obtained from short-term *ex vivo* expanded EBV/BMFL1-specific bulk T cell populations at T_n_ and T_n+15y_. **(D)** Data are representative of pooled dissociation rate (k_off_) values from 1 to 2 independent experiments. The mean value is indicated. **(E)** TCR-pMHC off-rate (k_off_) values from the first 40 to 60 *in vitro* generated EBV/BMFL1-specific, TRBV-unselected T cell clones at T_n_ and T_n+15y_ (BCL2, BCL9). For each *in vitro* single-cell cloning, T_n_ and T_n+15y_ samples were performed alongside. **(D, E)** p-values by Mann-Whitney test with p > 0.05, not significant [**(B)** (panel BCL9) and **(E)**] Data are depicted as box (25^th^ and 75^th^ percentiles) and whisker (min to max) plots with the middle line indicating the median.

We hypothesized that the preferential enrichment at late time-points with T cell clonotypes expressing low TCR binding avidity for CMV/pp65 but not for EBV/BMFL1 would lead to an overall decline in TCR repertoire avidity over time. To address this question, we aimed at directly measuring global k_off_-rates of polyclonal populations, similarly to a recent study (49). To do so, FACS-sorted virus epitope-specific CD8 T cells were rapidly expanded following short-term *in vitro* stimulation in order to reach sufficient cell numbers for the NTAmer assay, without introducing any major bias in the TRBV frequency distribution (**Supplementary Figures 4A, B**). Faster dissociation rates (i.e. lower k_off_) were observed on CMV/pp65-specific bulk T cells at the later time-point (i.e. T_n+15y_), revealing a significant decrease in the overall TCR binding avidity over time in three out of four donors (**Figures 4A, B**). Off-rates from BCL9 samples were highly heterogenous, due to the presence of two distinct NTAmer staining-based dissociation sub-populations, preventing accurate dissociation fitting and computation. Nonetheless, when we compared the proportion of CMV-specific T cells representative of the slower dissociation curves between time-points (**Figure 4A**, see FACS-gated region), there was as well a significant decline in the percentage of these slow-dissociating cells over time (**Figure 4B**).

Compared to the CMV/pp65 model, the overall off-rates of EBV/BMFL1-specific *ex vivo* generated bulk T cell populations showed a higher stability in k_off_-rates between T_n_ and T_n+15y_ with an absence of overall avidity loss (**Figures 4C, D**). Moreover, in BCL2 and BCL9, we assessed NTAmer-based k_off_ on a large panel of *in vitro* generated EBV-specific CD8 T cell clones for each time-point without further clonotype characterization or selection, thus representing an unbiased TCRαβ clonotype repertoire, and confirmed no significant differences in their global TCR-pMHC off-rates over time (**Figure 4E**). Similar findings were made when we reconstituted the overall repertoire avidity of each EBV-positive donor by pooling single clonotype-derived k_off_ measurements, obtained for most TCRαβ clonotypes, according to their respective prevalence (**Supplementary Figure 4C**). Collectively, these observations indicate that the TCR repertoires evolved towards an overall avidity decline during CMV (i.e. pp65_495-503_ epitope), but not EBV (i.e. BMFL1_280-288_ epitope) latency.

### Preferential LILRB1 expression in CMV/pp65-specific T cell clonotypes of high avidity

**Figure 5:**
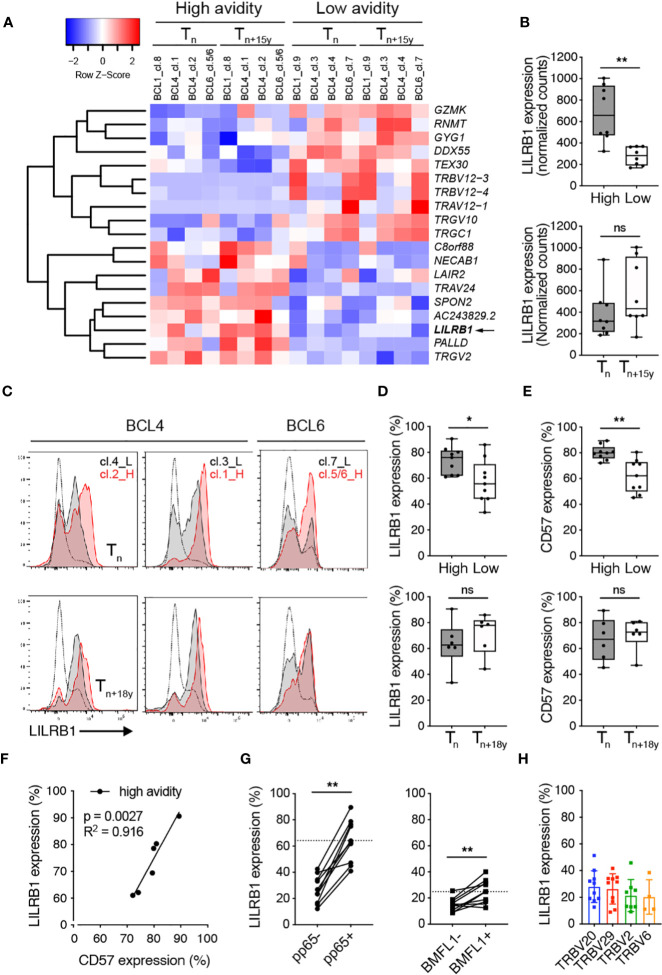
*Ex vivo* LILRB1 expression on CMV/pp65-specific TCRαβ clonotypes of high versus low avidity. **(A)** Heatmap plot based on the 19 differentially expressed genes (FDR < 0.1) between high- and low-avidity CMV/pp65-specific clonotypes obtained following *ex vivo* FACS-sorting using multimer/pp65- and TRBV-specific staining combined to RNA sequencing from BCL4, BCL6 and BCL1 at T_n_ and T_n+15y_. Red indicates over- and blue indicates under-expression relative to mean gene expression. **(B)** Direct comparison of *LILRB1* mRNA expression (as normalized counts) between high- and low-avidity CMV/pp65-specific clonotypes (top panel, n = 8) and at T_n_ and T_n+15y_ (bottom panel, n = 8). **(C)** Representative FACS-based histograms of *ex vivo* LILRB1 expression on high- and low-avidity CMV/pp65-specific clonotypes from BCL4 and BCL6 at T_n_ and T_n+18y_. For each single staining, LILRB1 expression on global CMV/pp65-negative CD8 T cells is depicted alongside (as gray curves). Note that for BCL6, TRBV6-5 staining includes two high avidity clonotypes (clono 5 and clono 6), whereas TRBV12 is only specific for the low avidity clono 7 (see **Figure 1C**). L, Low avidity; H, High avidity. **(D, E)** Frequencies of LILRB1 [**(D)**, n = 6-9] and CD57 [**(E)**, n = 6-9] surface expression on low- versus high-avidity CMV/pp65-specific clonotypes (top panels) and at T_n_ versus T_n+18y_ (bottom panels) from BCL4 and BCL6. Data are representative of 3 independent experiments. **(B, D, E)** Data are depicted as box (25^th^ and 75^th^ percentiles) and whisker (min to max) plots with the middle line indicating the median. p-values by Mann-Whitney unpaired test with ^*^p < 0.05 and ^**^p < 0.01, ns, non-significant. **(F)** Correlation between LILRB1 and CD57 surface expression on high-avidity CMV/pp65-specific T cell clonotypes from BCL4 and BCL6 (n = 6). Coefficient R^2^ and p-values from simple linear regression analyses are indicated. **(G)** Frequencies of LILRB1 expression on CMV/pp65- versus CMV/pp65+ CD8 T cells or EBV/BMFL1- versus EBV/BMFL1+ CD8 T cells from 10 healthy individuals. The dotted line indicates the mean values of LILRB1 expression found within pp65+ or BMFL1+ CD8 T cells, respectively. p-values by Wilcoxon non-parametric paired test with ^**^p < 0.01. **(H)** Frequency of LILRB1 expression on EBV/BMFL1-specific T cells according to their preferential TRBV family usage (n = 10).

To gain further insight into the mechanisms underlying the selection of low avidity T cell clonotypes during chronic CMV infection, we performed a global transcriptome profiling by RNA-Seq on *ex vivo* sorted CMV-pp65/TRBV-specific sub-populations at T_n_ and T_n+15y_ (**Figure 5A**; **Supplementary Figure 5A**). Hierarchical clustering highlighted the presence of 9 genes that were found enriched in clonotypes of high TCR binding avidity when compared with those of low avidity (**Figure 5A**). Among them, we identified *LILRB1* encoding for an inhibitory receptor (also known as CD85j or ILT2/LIR-1), associated in CD8 T cells with aging and CMV latent infection (50). *LILRB1* was significantly upregulated in high avidity CMV-specific clonotypes (**Figure 5B**). These data were validated by the NanoString technology (**Supplementary Figure 5B**). In addition, CMV/pp65-specific T cell clonotypes of high avidity presented enhanced surface levels of LILRB1 (**Figures 5C, D**) as well as of CD57 (**Figure 5E**), a marker of CD8 T cell differentiation, when compared to the low avidity ones. Hence, we observed, for the high avidity clonotypes, a positive correlation between LILRB1 and CD57 expression (**Figure 5F**). In agreement with previously documented reports (51–53), LILRB1 expression further displayed a progressive increase along CD8 T cell differentiation with highest levels found in effector CMV/pp65-specific T cells of high avidity (**Supplementary Figure 5C**). Finally, the frequency of LILRB1 expression on *ex vivo* CMV/pp65-specific T cells strongly exceeded that of EBV/BMFL1-specific T cells (**Figure 5G**). The latter cells expressing LILRB1 only at low frequencies, independently of their preferential TRBV family usage (**Figure 5H**; **Supplementary Figure 5D**) and consistent with a previous study (54).

### LILRB1 expression moderately restricts cell proliferation of CMV/pp65-specific T cell clonotypes of high avidity

**Figure 6:**
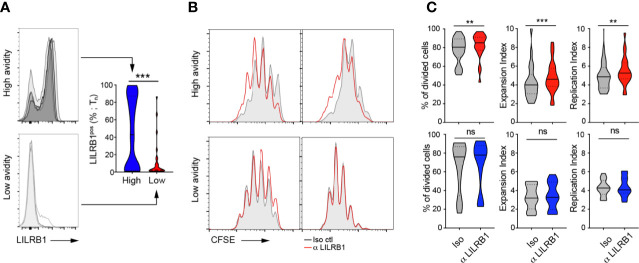
Blocking LILRB1 binding on *in vitro* cell proliferation of CMV/pp65-specific CD8 TCRαβ clonotypes. **(A)** Representative FACS-based histograms of LILRB1 expression on a representative selection of high- (top panel) and low- (bottom panel) avidity CMV/pp65-specific T cell clones at T_n_. Compiled data of LILRB1 expression levels (in percentage; right panel), depicted as violin plots (n = 27 to 61 T cell clones). p-values by Mann-Whitney test (two-tailed) with ^***^p < 0.001. **(B)** Representative overlays of CFSE histograms obtained from CFSE-labelled T cell clones of high avidity (top panels) or low avidity (bottom panels) in the presence of blocking anti-LILRB1 (red line) or an isotype control antibody (grey histogram) at day 4 after CMV/pp65-specific stimulation. **(C)** CFSE-based analyses (% of divided cells, expansion index and replication index) of high (top panels) or low (bottom panels) avidity CMV/pp65-specific CD8 T cell clones, treated with blocking anti-LILRB or an isotype control antibody. Data are depicted as violin plots (n = 10 to 22). p-values by Wilcoxon matched-pairs signed rank test with ^**^p < 0.01 and ^***^p < 0.001; ns, non-significant.

We next evaluated the biological significance of LILRB1 on cell proliferation (by CFSE) of CMV/pp65-specific T cell clonotypes from BCL4 upon direct LILRB1 blockade and *ex vivo* short term A2/pp65_495-503_ peptide-specific stimulation. Treatment with a blocking monoclonal antibody to LILRB1 (51) only induced a modest shift of the single CFSE dilution peaks obtained for clono 2 but not clono 1 high avidity T cells (**Supplementary Figure 6**). We further conducted CFSE quantification analyses after LILRB1 blockade and pp65 stimulation using a large panel of high versus low avidity *in vitro*-generated CMV/pp65-specific T cell clones. Actually, T cell clones of high avidities expressed significantly greater levels of LILRB1 than low avidity ones (**Figure 6A**). LILRB1 levels in high-avidity T cell clones were also enhanced when compared to the *ex vivo* LILRB1 expression data (**Figure 5**), likely resulting from the binding interactions occurring between LILRB1 and MHC class-I molecules (55) during the *in vitro* cultures of virus-specific CD8 T cells. LILRB1 blockade moderately improved the proliferation capacity (i.e. frequency of divided cells, expansion index and replication index) of high avidity but not that of low avidity T cell clones (**Figures 6B, C**). Despite the technical limitations linked to this functional assay, our data are in line with previously reported findings (51).

### Progressive *in vivo* loss of CMV/pp65 T cell clonotypes of high avidity is associated to enhanced LILRB1 expression

**Figure 7:**
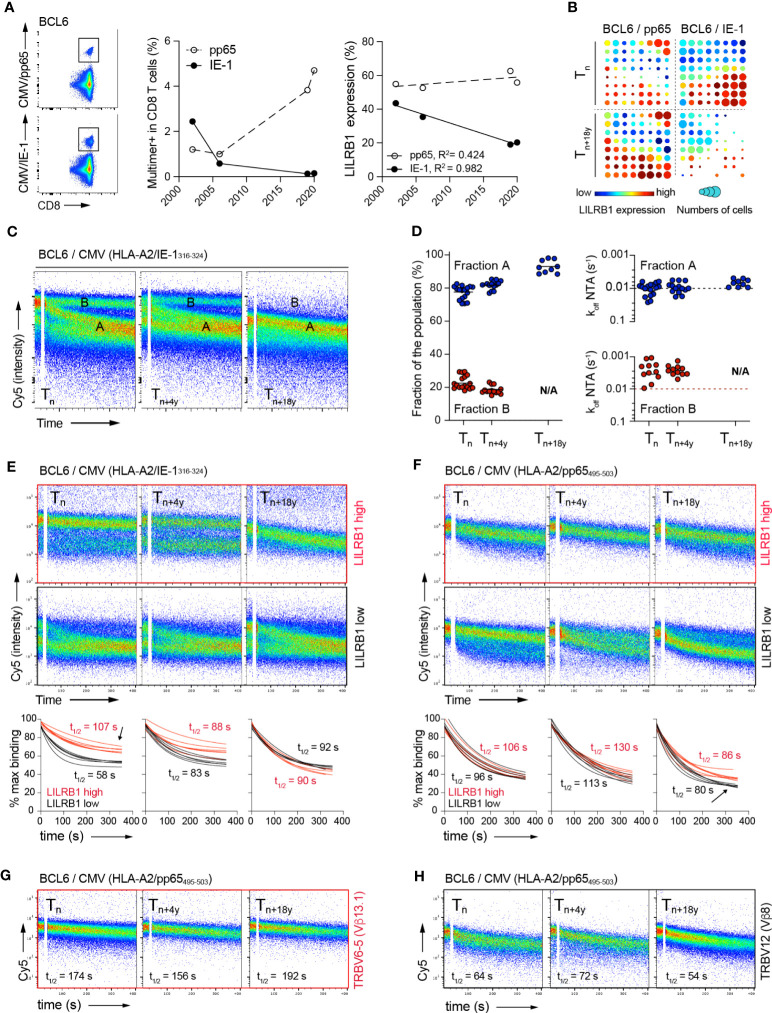
Relationship between LILRB1 expression, TCR binding avidity and *in vivo* persistence of CMV-specific CD8 T clonotypes over time. **(A)** Representative FACS analysis of CMV/pp65 (top panel) or CMV/IE-1 (bottom panel) multimer staining on CD8 T cells from donor BCL6. Evolution of CMV/pp65 and CMV/IE-1 multimer-positive fractions (middle panel) and LILRB1 expression within CMV/pp65 or CMV-IE-1-specific CD8 T cells (right panel) over time (2002 to 2020). **(B)** T-SNE analysis & FlowSOM visualization at T_n_ and T_n+18y_ of merged events from CMV/pp65- (n = 2348 cells) and CMV/IE-1- (n = 2898 cells) specific CD8 T cells from BCL6. On the FlowSOM grid plots, each cluster is sized proportionally to the fraction of multimer-positive CD8 T cells. The cluster’s color represents LILRB1 mean expression from low (blue) to high (red). **(C)** Representative FACS-based NTAmer dissociation curves obtained from *ex vivo* expanded CMV/IE-1-specific bulk populations at T_n_, T_n+4y_ and T_n+18y_. The population of cells with fast (fraction A) or slow (fraction B) dissociation rates, respectively are indicated. **(D)** Quantification of the CMV/IE-1-specific CD8 T cells present within fraction A or B dissociating populations (n > 9 independent experiments, left panel). Dissociation rate (k_off_) values derived from the cell populations present within fraction A or fraction B at T_n_, T_n+4y_ and T_n+18y_ (right panel). **(E, F)** Combined NTAmer dissociation measurements and LILRB1 costaining on *ex vivo* expanded CMV/pp65- **(E)** and CMV/IE-1- **(F)** specific bulk T cells at the indicated time-points (T_n_, T_n+4y_ and T_n+18y_). Representative FACS-based wild-type NTAmer dissociation curves obtained after gating on LILRB1^high^ (top panels) versus LILRB1^low^ (bottom panels) expression. Compiled TCR-pMHC dissociation rates of LILRB1^high^ (in red) versus LILRB1^low^ (in black) subpopulations over time (n = 6). Arrows indicate the dissociating subpopulations of high-avidity and LIRB1^high^ expression (in red) within bulk CMV/IE-1-specific T cells at T_n_ (i.e. contracting cells) or of low-avidity and LILRB1^low^ expression (in black) within bulk CMV/pp65-specific T cells at T_n+18y_ (i.e. expanding cells), respectively. **(G, H)** Combined NTAmer dissociation measurements and TRBV costaining on *ex vivo* expanded CMV/pp65-specific bulk T cells at the indicated time-points (n = 2). Representative FACS-based wild-type NTAmer dissociation curves obtained after gating on TRBV6-5 [high avidity; **(G)**] versus TRBV12 [low avidity; **(H)**] clonotypes.

Since the NTAmer assay relies on flow-cytometry avidity analyses, it can be easily adapted to combine direct *ex vivo* phenotype and off-rate measurements alongside. Studies were further performed on the two immune-dominant CMV/A2 CD8 T cell epitopes (i.e. CMV/pp65_495-503_ versus CMV/immediate-early (IE)-1_316-324_) only detectable in donor BCL6 (**Figure 1B**). In this healthy individual, CMV/pp65-specific T cells accumulated during the 18 years of follow-up, whereas CMV/IE-1-specific T cells showed a gradual decline in frequency over time, concomitant to a decrease in LILRB1 expression (**Figure 7A**). These observations were confirmed using dimensionally reduction t-SNE and FlowSOM clustering analysis (38). At T_n_, LILRB1 was expressed in a large fraction of the CMV/IE-1-specific T cells, whereas this distinct sub-population had almost vanished at T_n+18y_ (**Figure 7B**). This was not the case for CMV/pp65-specific T cells with an overall preserved LILRB1 expression over this time-frame (**Figures 7A, B**).

We next assessed TCR-pMHC dissociation rates at the *ex vivo* CMV/IE-specific T cell population level. This analysis revealed the presence of several dissociation curves, arbitrarily defined as fraction A (i.e. faster off-rates) and fraction B (i.e. slower off-rates) (**Figure 7C**). Fraction A represented the predominant subpopulation at T_n_, which further prevailed during the course of latent infection, while retaining relatively stable off-rates (**Figure 7D**). In contrast, fraction B, mostly composed of T cells of highest binding avidities, had entirely disappeared at T_n+18y_. These data indicate that in donor BCL6, CMV/IE-specific T cells not only contracted during latent infection, but this process was associated with the preferential loss of high avidity T cell repertoires, in line with the data found for the CMV/pp65 model (**Figure 4**). Finally, we conducted a NTAmer-based off-rate analysis combined to LILRB1 and TRBV co-staining. Strikingly, LILRB1 expression corresponded to the fraction of IE-1-specific T cells of higher TCR binding avidity/slower off-rates at T_n_, which was no longer detectable at T_n+18y_ (**Figure 7E**, see arrow). Despite conserved LILRB1 expression over time within CMV/pp65-specific T cells (**Figures 7A, B**), we further observed the gradual appearance of a subpopulation of lower avidity/faster off-rates, characterized by reduced LILRB1 levels (**Figure 7F**, see arrow) and by the presence of TRBV12 clonotype of known low binding avidity (**Figures 7G, H**; **Supplementary Figure 7**). Collectively, these observations indicate a progressive *in vivo* loss of high avidity CMV-specific CD8 T cells with time, corresponding to T cells expressing increased LILRB1 levels.

## Discussion

Here, we investigated whether TCR-ligand avidity can directly drive the long-term maintenance of particular herpesvirus-specific CD8 TCRαβ clonotypes and searched for factors that regulate clonal repertoires in latent human CMV versus EBV infection, over a follow-up time of 15-18 years. Our data revealed the progressive *in vivo* loss of CMV/pp65- and CMV/IE-1-specific T cell clonotypes of high binding avidity (i.e. slow TCR off-rates, reduced CD8 binding dependency) during long-term antigen exposure. This was not the case for EBV/BMFL1-specific CD8 T cell repertoires, in which the clonal composition and distribution (i.e. dominant versus sub-dominant, slow versus fast TCR off-rates, CD8 binding-independent versus -dependent) are kept highly stable for at least 15 years. Together, these findings indicate that TCR-pMHC binding avidity is a determining factor driving the clonal evolution of long-lasting CMV- but not EBV-specific memory CD8 T cell responses in humans. In addition, LILRB1 checkpoint receptor was preferentially expressed in high-avidity CMV-specific TCRαβ clonotypes and correlated in those cells to their gradual frequency decline over time. The main weakness of the study is the relative low number of available volunteers (n = 6), due to the exceptionally long period of follow-up and the fact that chronic CMV/pp65-specific T cell responses are typically composed of highly restricted co-dominant clonotypes (n = 15). Consequently, this somehow limits the strength of our statistical-based conclusions.

Differences in the nature of these β-herpesviruses during latent infection could in part explain the gradual contraction of high avidity CMV/pp65- and CMV/IE-1-specific T cell clonotypes observed over time, contrasting with the striking stability of EBV/BMFL1-specific CD8 T cell clonal repertoires. In the context of EBV infection, some infected B cells become long-lived, namely the latently infected memory B cells (16). EBV reactivation and replication in B cells occurs only sporadically, leading to intermittent cycles of T cell rest and stimulation, in contrast to CMV, which is considered more as a smoldering latent/chronic infection (16, 56). In fact, there is growing evidence that CMV undergoes low-level viral replication, which potentially impacts the virus-specific CD8 T cell responses (15), with the induction of large populations of highly functional T cell responses, maintained lifelong (14, 57). The latent CMV reservoir in MCMV is largely confined to non-hematopoietic cells, such as lymphatic endothelial cells, and can selectively induce memory CD8 T cell inflation (22, 58). In addition, reactivation of CMV or EBV is commonly found in immunocompromised individuals after allogeneic hematopoietic stem cell transplantation (allo-HSCT). CMV has been described as the most frequently reactivated virus following allo-HSCT, occurring in 65-68% of the patients, contrasting to the 9-15% of patients developing EBV reactivation (59, 60). Altogether, these observations including ours further indicate differing mechanisms of viral latency and reactivation between these two herpesviruses. This is in line with a large, recent comprehensive profiling study of flu- versus CMV-specific T cells across multiple tissue sites of organ donors revealing that memory persistence and functional regulation to viral antigens is primarily shaped by virus tropism and specificity (61).

Schober and colleagues (62) proposed different theoretical models of TCR repertoire evolution during latent CMV infection, and to which degree this process is controlled by TCR-pMHC binding/structural avidity. One model hypothesizes an initial accumulation of high-avidity virus-specific T cells during the early phase of latency, followed by the succession of clones of lower TCR binding avidity over the course of latent infection (62, 63). During this clonal evolution, T cells of higher TCR binding avidity may progressively be lost after replicative senescence due to critical telomere length shortening (64, 65). In the context of MCMV infection, the same authors recently demonstrated that high-avidity TCRs dominated T cell responses at early time-points after infection, but then underwent clonal succession, cellular differentiation and senescence during late time-points, leading to a switch in dominance toward low-avidity TCR T cells (32). Our findings in humans are also compatible with this model, as we showed the preferential selection and expansion of CMV/pp65-specific clonotypes of lower TCR binding avidity, compared to those that had contracted after 15-18 years. Consequently, an overall avidity decline over time was observed at the CMV/epitope-specific population (i.e. HLA-A2/pp65_495-503_ and HLA-A2/IE-1_316-324_). As shown here in humans, longer observation periods (i.e. >15-20 years) are required to reveal the contraction of high-avidity CMV/epitope-specific clones in the latency infection phase, as T cell repertoires can be maintained relatively stable during at least several years (40, 44). A recent report, using adoptive transfer of low versus high avidity MCMV-specific CD8 T cell subpopulations, revealed that during the early phase of latent infection, the inflationary T cell pool was comprised mainly of high avidity CD8 T cells, outcompeting lower avidity CD8 T cells (66). These discordant results could be explained by the use of high number of transferred cells and a relatively short period of follow-up. In summary, our extended longitudinal analysis further argues for an intrinsic impact of the TCR-pMHC avidity on repertoire evolution over the course of latent CMV infection (32, 62).

The remarkable stability in the clonal evolution of EBV-specific repertoires raises the question whether this represents a global characteristic of EBV-specific CD8 T cell responses or only of responses against the EBV epitope studied here (i.e. HLA-A2/BMFL1_280-288_). Since HLA-A2/BMFL1_280-288_ is an epitope from the early protein BMFL1 essentially expressed during the acute phase of infection, the stability of the observed clonotype repertoires over extended periods of time could potentially be explained by the relative absence of epitope expression during the latent phase. Nonetheless, TCR clonotype composition and distribution against HLA-A2/BMFL1_280-288_ were also highly preserved during transient immunological perturbations after non-myeloablative chemotherapy (44) or following lung transplant under immunosuppression in the presence of EBV reactivation (46). Moreover, long-lasting dominance of EBV-specific TCRαβ clonotypes against two latent epitopes, with no changes in the T cell hierarchy for at least 18 years has been previously reported (67). Collectively, these observations, including our own study, are consistent with the concept that TCRαβ clonotype responses against EBV, once established, show steady repertoires over these long periods.

Memory inflation describes the longitudinal development of stable, expanded CD8 T cell memory pools with a distinct phenotype and functional profile, but without sharing features of immune exhaustion (14). Whereas memory CD8 T cell inflation is a hallmark of CMV infection in mouse models, this process is less clearly defined in human CMV infection (68). This is probably due to (i) the large inter-individual variability including notably the duration of infection, HLA restriction, size of latent virus pool, and recurrence of viral reactivation events as well as (ii) the paucity of long-term longitudinal studies (57). In this regard, data from a recent 27-year-long longitudinal study showed only limited effect of duration of CMV infection on adaptive immunity and frailty (69). With the exception of donor BCL6, we did not observe a significant increase in CMV/pp65-specific CD8 T cell frequencies over the observation period of 15-18 years. Moreover, this highly contrasted to the drastic over time decline of CMV/IE-1-specific T cells in BCL6, the only donor sharing multiple specificities. Nevertheless, we still observed a progressive loss of T cells of high binding avidity, with an enrichment of low avidity T cells, in both immunodominant epitopes. Accordingly, we did not observe an inverse relationship between population size and TCR-pMHC half-lives, as recently illustrated for MCMV-specific T cell responses (32). It is possible that our study performed on middle-aged individuals (45 +/- 10 years) was still set too early in the course of the latent phase to visualize CMV-specific T cell expansion, or that memory inflation remains hard to be studied even in well-designed longitudinal studies of healthy individuals, due to the above-mentioned confounding factors (57).

As other inhibitory receptors, LILRB1 (also known as CD85j or ILT2/LIR-1) is frequently found on the surface of human CD8 T cells (70). This is the case for highly differentiated antigen-experienced T cells, often expressing CD57 and lacking CD28, while displaying strong effector functions (51–53). The proportion of LILRB1-positive T cells has been shown to increase with age and in chronic CMV infection (50, 51). LILRB1 recognizes with high affinity HLA-G and UL18, a human CMV-encoded MHC class I homologue and with lower affinity classical MHC class I molecules (71). Importantly, interactions between inhibitory leukocyte Ig-like receptors, including LILRB1, and endogenous and pathogenic ligands can regulate both innate and adaptative immune responses, leading to tolerance and immune evasion (72). Specifically, LILRB1 expressed on CD8 T cells can inhibit immune cell activation and effector function (51–53, 73–75). Therefore, it has been proposed that LILRB1 functions as an immunosuppressive receptor and may represent an attractive target to enhance tumor cell killing by effector CD8 T cells (52, 53, 72–74). Extending on these observations, we found that LILRB1 was preferentially expressed on CMV-specific TCRαβ clonotypes of high avidity compared to the low avidity ones and may potentially contribute to their gradual *in vivo* loss over time during latent CMV infection. This contrasted to the low, steady LILRB1 expression observed in EBV-specific CD8 TCRαβ clonotypes, unrelated to their TCR-pMHC binding avidity. Our data are further in line with the previous observations that latent CMV infection drives stronger T cell differentiation and clonal expansion than the EBV one, resulting in clonal diversity loss (5–10). Interestingly, Schober and colleagues (32) recently showed that MCMV-specific T cell clones that declined during the chronic infection phase were also those of higher TCR avidity and underwent cellular senescence without displaying signs of classical exhaustion.

Robust techniques allowing for the large-scale *ex vivo* assessment of TCR-pMHC binding kinetics at the surface of live T cells have proven technically challenging until recently (76). Using fluorescent reversible NTAmers, we previously showed that the k_off_ parameter represents a powerful biomarker by which the functional potency of antigen-specific CD8 T cell responses can be directly evaluated (33, 34) and graded to better characterize their impact on the efficacy of therapeutic vaccines (77). Here, we used NTAmers on well-identified virus-specific TCRαβ clonotypes or at the polyclonal CD8 T cell population level and observed for the first time a differential impact of TCR-pMHC binding kinetics on the long-term TCR clonal evolution during CMV (i.e. pp65 and IE-1) versus EBV/BMFL1 latent infection. Moreover, comparable TCR-ligand off-rate (k_off_) values were found for all studied representatives of a given CMV- or EBV-specific clonotype over time (T_n_ versus T_n+15y_), indicating that TCR-pMHC dissociation rate represents a stable intrinsic biometric at the TCRαβ clonotype level. Our findings nicely fit with another recent report performed on MCMV-specific TCR repertoires during the course of infection using TCR-pMHC k_off_-rate measurements with reversible *Strep*tamers (32). Moreover, the NTAmer technology is based on reversible peptide-MHC multimers and real-time flow cytometry, allowing to directly combine TCR-pMHC off-rate analysis to LILRB1 and specific TRBV staining. Using this upgraded approach, we demonstrated a direct relationship between CMV epitope-specific TCR binding avidity and LILRB1 expression. Specifically, our results suggest a selective expansion of low-avidity LILRB1^low^ T cells, outcompeting high-avidity LILRB1^high^ T cells. Alternatively, high-avidity LILRB1^high^ T cells may directly be depleted over time as shown for the CMV/IE-1 epitope. Either way, TCR avidity-based repertoire evolution may further be described as a “clonal succession model”, in which T cells of relative higher avidity are continuously being replaced over time by the next generation of lower avidity T cells, in a constant and dynamic recruitment process.

## Conclusive remarks

Our study reinforces the key role driven by TCR-ligand binding avidity in tailoring CMV- but not EBV-specific clonal evolution during long periods of viral latency. We further propose that LILRB1 may act as an inhibitory checkpoint receptor, by potentially limiting the expansion of high avidity T cell clonotypes over the course of latent CMV infection. In the context of CMV infection, repetitive exposure to antigen is a key determinant for memory expansion/inflation, and therefore regulatory mechanisms that can repress CD8 T cell expansion are likely beneficial (15). Such mechanisms include regulatory T cells and anti-inflammatory cytokine production, which might otherwise lead to the extensive proliferation of highly antigen-sensitive T cells, possibly overwhelming the global T cell pool (57). Supporting this notion, LILRB1 may therefore provide another mechanism by which memory expansion of given CMV-specific TCRαβ clonotypes might be tightly regulated during lifelong latent/reactivating CMV infection, while preserving the global functional T cell repertoire.

## Data availability statement

The data presented in this study are deposited in the GEO repository, accession number GSE246111.

## Ethics statement

The studies involving humans were approved by the Ethics Committee for Clinical Research of the University of Lausanne (Lausanne, Switzerland). The studies were conducted in accordance with the local legislation and institutional requirements. The participants provided their written informed consent to participate in this study.

## Author contributions

BC: Conceptualization, Data curation, Formal Analysis, Methodology, Writing – original draft. BD: Formal Analysis, Methodology, Writing – review & editing. LC-I: Formal Analysis, Methodology, Writing – review & editing. MA: Formal Analysis, Methodology, Writing – review & editing. SP: Formal Analysis, Methodology, Writing – review & editing. MH: Conceptualization, Formal Analysis, Investigation, Methodology, Supervision, Validation, Writing – review & editing. NR: Conceptualization, Formal Analysis, Funding acquisition, Methodology, Project administration, Resources, Supervision, Validation, Writing – original draft, Writing – review & editing.
