# Supplementary material for: Overall avidity declines in TCR repertoires during latent CMV but not EBV infection
Source: Front Immunol. 2023 Nov 20;14:1293090. doi: 10.3389/fimmu.2023.1293090 (PMC10694213; doi:10.3389/fimmu.2023.1293090)
Supplement: Supplementary file 1 [file DataSheet_1.pdf]

SUPPLEMENTARY DATA

SUPPLEMENTARY MATERIALS & METHODS

Ex vivo global transcriptome profiling by RNA sequencing analysis

Purity-filtered reads were adapters and quality trimmed with Cutadapt (v.1.8, (1)). Reads matching to ribosomal RNA sequences were removed with fastq_screen (v.0.11.1). Remaining reads were further filtered for low complexity with reaper (v.15-065, (2)). Reads were aligned against Homo sapiens.GRCh38.92 genome using STAR (v.2.5.3a, (3)). The number of read counts per gene locus was summarized with htseq-count (v.0.9.1, (4)) using Homo sapiens.GRCh38.92 gene annotation. Quality of the RNA-seq data alignment was assessed using RSeQC (v.2.3.7, (5)). The effect TCR avidity and time were tested in R (v.3.4.0) using the likelihood ratio test implemented in the DESeq2 package (6). A linear model with the TCR binding avidity (low and high avidity), the time-point (2002, 2017) and the donors (BCL1, BCL4, BCL6) was compared to reduced models with (i) the TCR avidity factor removed or (ii) the time-point factor removed. Parameters 'cooksCutoff' and 'independentFiltering' were set to 'false'. The gene expression data have been deposited in the NCBI Gene Expression Omnibus (GSE246111).

NanoString analysis

Total RNA from CMV-specific CD8 T cell clonotypes of high versus low TCR binding avidity was extracted using the RNeasy Mini Kit (Qiagen) according to the manufacturer's protocol. The NanoDrop spectrophotometer (Thermo Scientific) was used to quantify the RNA. Cell lysates were directly analyzed for the expression of 770 immune-related genes (human PanCancer immune profiling panel) by the NanoString nCounter System and data were further processed using the NanoString nSolver analysis software (v.4) by normalizing with housekeeping genes.

Ex vivo LILRB1 and CD57 expression along T cell differentiation

To assess CD57 and LILRB1 expression along T cell differentiation, CMV/pp65-specific CD8 T cell clonotypes of high versus low avidity isolated and stained as described in the main manuscript (CD8, CD28, CD45RA, CD57, CD85j, CMV/pp65 multimer, Vivid Aqua and appropriate TRBV antibodies (**Supplementary Table 1**)) were acquired on a Cytoflex flow cytometer (Beckman Coulter) and data were imported in FlowJo software (v.10.4.2, Tree Star) for analysis, using both CD28 and CD45RA as differentiation markers along which LILRB1 and CD57 positive cells were quantified as fractions of parent population.

Ex vivo LILRB1 blocking experiments

CFSE-labelled (Invitrogen) PBMCs from donor BCL4 were pulsed 1 hour at 37°C with native CMV/pp65$_{495\text{-}503}$ peptide (10^{-8} M) washed and cultured in RPMI 1640 medium (Gibco) supplemented with 8% human serum and 50 U/ml human recombinant IL-2 (GlaxoSmithKline) in the presence of monoclonal IgG$_{2B}$ mouse anti-human LILRB1 (CD85j, ILT-2) blocking antibody or an IgG$_{2B}$ isotype control used at 5 µg/ml (R&D Systems). After 3-6 days, cells were washed, stained with Near-IR Vivid (Invitrogen), CMV/pp65-specific multimers, anti-CD8 antibodies (Beckman Coulter) and anti-TRBV antibodies specific for the TCRαβ clonotypes identified in BCL4 (**Supplementary Table 1**) before acquisition on a LSRII (BD Biosciences) cytometer. The percentage of divided cells, as well as the expansion and replication indexes were determined using the proliferation analysis tools available in FlowJo (v.10.4.2, Tree Star)

Supplementary Table 1. List of TRBV antibodies used in the study

Name*	IMGT nomenclature	Company	Catalog #	Clone
Pe-Vio770 anti-Vβ1	TRBV9	Miltenyi	130-110-020	REA662
APC-Vio770 anti-Vβ2	TRBV20	Miltenyi	130-110-098	REA654
FITC anti-Vβ3	TRBV28	Beckman Coulter	IM2372	CH92
FITC anti-Vβ4	TRBV29	Beckman Coulter	B07084	WJF24
FITC anti-Vβ8	TRBV12	Beckman Coulter	IM1233	56C5.2
APC anti-Vβ9	TRBV3	Invitrogen	17-4899-41	AMKB1-2
FITC anti-Vβ13.1	TRBV6-5	Invitrogen	11-5792-41	H131
APC anti-Vβ14	TRBV27	Miltenyi	130-108-738	REA557
APC anti-Vβ16	TRBV14	Miltenyi	130-108-769	REA553
FITC anti-Vβ22	TRBV2	Beckman Coulter	IM1484	IMMU546

*TCR Vβ according to Arden's nomenclature

Supplementary Table 2. List of CMV/pp65-specific TCRαβ clonotypes

Donor	Clonotype	IMGT	BV Arden	CDR3β	BJ Arden	IMGT	AV Arden	CDR3α	AJ Arden	TCR avidity
**BCL4**	Clono 1	TRBV27	14	RLLAGGRSAQ	2.5	TRAV24	18	EGGNQF	49	High
**CMV**	Clono 2	TRBV3-2	9S2	SLLLGTAAEA	1.1	TRAV24	18	IAGNQF	49	High
	Clono 3	TRBV9	1	SVYGGAGNSPL	1.6	TRAV14	6	KNFNKF	21	Low
	Clono 4	TRBV28	3	SFLGYTEA	1.1	TRAV3	16	YYGQNF	26	Low
**BCL6**	Clono 5	TRBV6-5	13S1	SRQTGAAYGY	1.2	TRAV24	18	NTGNQF	49	High
**CMV**	Clono 6	TRBV6-5	13S1	SYATGTAYGY	1.2	TRAV24	18	NTGNQF	49	High
	Clono 7	TRBV12	8S1/2	SSANYGY	1.2	TRAV35	25	PRETSYDKV	50	Low
**BCL1**	Clono 8	TRBV9	1	SVVGLWTDTQ	2.3	TRAV14	6	PMKTSYDKV	50	High
**CMV**	Clono 9	TRBV12	8S1/2	SSANYGY	1.2	TRAV35	25	EPENSGGSNYKL	53	Low
**BCL9**	Clono 10	TRBV7-3	6	SLMALGAGANVL	2.6	na	na	na	na	High
**CMV**	Clono 11	TRBV28	3	SFQGYTEA	1.1	na	na	na	na	High
	Clono 12	TRBV9	1	SPLGGAGLADTQ	2.3	TRAV14	6	REGIIQGAQKL	54	Low
	Clono 13	TRBV27	14	SLTPGSPGSPL	1.6	na	na	na	na	High
	Clono 14	TRBV6-5	13S1	SPTTGTGYFGY	1.2	TRAV24	18	NTGNQF	49	High
	Clono 15	TRBV6-5	13S1	SLVSGSGSYGY	1.2	TRAV24	18	NTGNQF	49	High

Supplementary Table 3. List of EBV/BMFL1-specific TCRαβ clonotypes

Donor	Clonotype	IMGT	BV Arden	CDR3β	BJ Arden	IMGT	AV Arden	CDR3α	AJ Arden
BCL4 EBV	2 clono 1	TRBV20	2	RDRIGNGY	1.2	TRAV5	15	DNNARL	31
	2 clono 2*	TRBV20	2	RDRTGNGY	1.2	TRAV5	15	DNNARL	31
	2 clono 3	TRBV20	2	RDSVGNGY	1.2	TRAV5	15	DNNARL	31
	2 clono 4	TRBV20	2	RDRVGNGY	1.2	TRAV5	15	DNNARL	31
	2 clono 5	TRBV20	2	RDSTGNGY	1.2	TRAV5	15	DNNARL	31
	4 clono 1	TRBV29	4	FQEASYGY	1.2	TRAV29	21	SGGSQGNL	42
	4 clono 2**	TRBV29	4	VGTGGTNEKL	1.4	TRAV5	15	STGKL	37
	4 clono 3	TRBV29	4	VGYGGTNEKL	1.4	TRAV5	15	STGKL	37
	4 clono 4	TRBV29	4	VGSGGTNEKL	1.4	TRAV5	15	DNNARL	31
	4 clono 5	TRBV29	4	TPGQLMETQ	2.5	TRAV5	15	TLGNTGKL	37
	16 clono 1	TRBV14	16	SQSPGGTQ	2.5	TRAV5	15	SPPSSASKI	2
	18 clono 1	TRBV18	18	SPPAVSYEQ	2.7	TRAV29	21	IHNQAGTAL	15
BCL7 EBV	2 clono 1	TRBV20	2	RDTIGNGY	1.2	TRAV5	15	DNNARL	31
	2 clono 2***	TRBV20	2	RVGVGNTI	1.3	TRVA5	15	DNNARL	31
	2 clono 3	TRBV20	2	RDRVGNTI	1.3	TRAV5	15	DQSPRV	31
	2 clono 4*	TRBV20	2	RDRTGNGY	1.2	TRAV5	15	DNNARL	31
	2 clono 5	TRBV20	2	RSETGNTI	1.3	TRAV5	15	DNNARL	31
	2 clono 6	TRBV20	2	RGSVGNTI	1.3	TRAV5	15	DSNARL	31
	2 clono 7	TRBV20	2	RIGVGNTI	1.3	TRAV5	15	DNNARL	31
	2 clono 8	TRBV20	2	RDRVGNGY	1.2	TRAV5	15	DVNARL	31
	2 clono 9	TRBV20	2	RDETGNGY	1.2	TRAV5	15	DNNARL	31
	2 clono 10	TRBV20	2	WDREVMGGNTI	1.3	TRAV5	15	TSSASKI	3
	4 clono 1	TRBV29	4	VGSGGTNEKL	1.4	TRAV5	15	SIGKL	34
	4 clono 2	TRBV29	4	TTGSGDRGA	1.1	TRAV5	15	DRYSTL	11
	4 clono 3	TRBV29	4	VEGLTYNEQ	2.1	TRAV12-2	2S1	ITGGTYKY	40
	4 clono 4	TRBV29	4	VGEGGTNEKL	1.4	TRAV5	15	SIGKL	37
	4 clono 6**	TRBV29	4	VGTGGTNEKL	1.4	TRAV5	15	STGKL	37
	22 clono 1	TRBV2	22	TSGQISPSAI	1.3	TRAV12-1	2S3	NGGDSSYKL	12
	22 clono 2	TRBV2	22	TAGGTLPGEQ	2.7	TRAV12-1	2S3	NGMDSSYKL	12
	22 clono 3	TRBV2	22	SGGQVAPSEQ	2.1	TRAV12-1	2S3	NGEDSSYKL	12
	22 clono 4	TRBV2	22	SSGSVAPGEL	2.2	TRAV12-1	2S3	NGRDSSYKL	12
	22 clono 5	TRBV2	22	SSLEVSPSEQ	2.7	TRAV12-1	2S3	NGKDSSYKL	12
	22 clono 6	TRBV2	22	TSGTVAPGEQ	2.7	TRAV12-1	2S3	NGMDSSYKL	12
BCL2 EBV	2 clono 1	TRBV20	2	RDEVSGSWNEQ	2.1	TRAV9-2	22	SGDRDDKI	30
	2 clono 2	TRBV20	2	RDQTGNGY	1.2	TRAV5	15	DNNARL	31
	2 clono 3	TRBV20	2	RDREFGNTI	1.3	TRAV5	15	DSNARL	31
	2 clono 4***	TRBV20	2	RVGVGNTI	1.3	TRAV5	15	DNNARL	31
	4 clono 1	TRBV29	4	VGTGGTNEKL	1.4	TRAV5	15	SVGKL	8
	4 clono 2	TRBV29	4	VEDSIAGFTDTQ	1.4	TRAV9-2	22	NGGFKT	9
	4 clono 3	TRBV29	4	VGQGGTNEKL	1.4	TRAV5	15	SSSVGY	40
	22 clono 1	TRBV2	22	SEGAVAPGEQ	2.7	TRAV12-1	2S3	NGMDSSYKL	12
	22 clono 2	TRBV2	22	TDPRLLPGEQ	2.7	TRAV12-1	2S3	NGADSSYKL	12
	22 clono 3	TRBV2	22	SVGEILPGEQ	2.7	TRAV12-1	2S3	NGRDSSYKL	12
	13 clono 1	TRBV6	13	KTGTGNEKL	1.4	TRAV5	15	PNNAGNML	39
	13 clono 2	TRBV6	13	PPMGTPNYGY	1.2	TRAV5	15	DNNARL	31
	13 clono 3	TRBV6	13	SEWTGYQPQ	1.5	TRAV29	21	SGGGADGL	45
BCL9 EBV	13 clono 1	TRBV6	13	TPDVNTEA	1.1	TRAV5	15	SMDGYAL	41
	16 clono 1	TRBV14	16	SQSPGGTQ	2.5	TRAV5	15	SRETAL	15
	16 clono 2	TRBV14	16	SQSPGGIQ	2.4	TRAV5	15	GGADGL	45
	22 clono 1	TRBV2	22	SPPGLAPNEQ	2.1	TRAV12-1	2	NGKDSSYKL	12
	22 clono 2	TRBV2	22	SGGRVAPGEL	2.2	TRAV12-1	2	NGRDSSYKL	12
	22 clono 3	TRBV2	22	SDGAVAPNEQ	2.1	TRAV12-1	2	NGRDSSYKL	12
	22 clono 4	TRBV2	22	SEGQVWPGEL	2.2	TRAV12-1	2	NGMDSSYKL	12
	6 clono 1	TRBV7-3	6	SSGGNIDTQ	2.3	na	na	na	na
	12 clono 1	TRBV10-3	12	KSFRHRYSE	2.3	TRAV5	15	DNNARL	31
	7 clono 1	TRBV4-2	7	SQDGAGGLGEQ	2.1	TRAV12-1	2	NIPNDYKL	20

*, **, ***; public TCRαβ clonotypes

SUPPLEMENTARY FIGURE LEGENDS

Supplementary Figure 1. Frequencies of TRBV family-based virus-specific CD8 T cells from healthy donors. (A) Frequencies of CMV (HLA-A2/pp65)- and EBV (HLA-A2/BMFL1)-specific cells within total *ex vivo* CD8 T cells from 6 donors (BCL4 and BCL9 contain both CMV/pp65 and EBV/BMFL1 specificities) at T_n and T_{n+15y}. **(B)** Representative FACS dot plots of *ex vivo* CMV/pp65-specific CD8 T cells from BCL4, BCL6, BCL1, and BCL9, labeled with the indicated anti-TRBV family antibodies at T_n. Percentages of positively stained cells are indicated. **(C)** Correlations of TRBV frequencies between direct *ex vivo* TRBV-family staining and *in vitro* single-cell cloning and *TRBV-CDR3* sequencing, obtained from CMV/pp65- (left panel; BCL4, BCL6, BCL1 and BCL9) and EBV/BMFL1- (right panel, BCL2, BCL7 and BCL9) specific CD8 T cells. Coefficient R^2 and p-values from simple linear regression analyses are indicated. **(D)** Quantification of TRBV-positive CMV/pp65-specific T cells obtained by *ex vivo* TRBV-family staining at T_n and T_{n+15y} for each healthy donor, adjusted to the respective CMV/pp65-specific population size (multimer$^+$CD8$^+$ T cells). Results are depicted as percentages of color-coded TRBV family frequencies, corresponding each to a unique TRBV-CDR3 clonotype. Except of BCL6/TRBV6-5 with two distinct clonotypes (clono 5 and clono 6, shown in gray), the same color barcodes as in main Figure 1D were used. The fraction of TRBV-unlabeled CMV/pp65-specific CD8 T cells is depicted in white.

Supplementary Figure 2. Monomeric TCR-pMHC dissociation rates of CMV/pp65-specific CD8 TCRαβ clonotypes over time. (A) Representative FACS-based wild-type NTAmer dissociation curves obtained for different CMV/pp65-specific TCRαβ clonotypes (clono 5, clono 6 and clono 7) from donor BCL6 at the indicated time-points. **(B)** TCR-pMHC dissociation rate (k_{off}) by CD8 mutated NTAmers (CD8null NTA) of each color-coded CMV/pp65-specific TCRαβ clonotype from BCL6 at the indicated time-points. CD8null NTA non-binder clones bearing the TCRαβ-specific clonotype 7 (i.e. clono 7) are represented in the gray boxes. This particular clonotype is defined as CD8 binding-dependent compared to CD8 binding-independent clonotypes, clono 5 and 6. **(C, D)** Correlations of CD8 null NTAmer-based TCR off-rates (CD8null NTA; k_{off}) between T_n and T_{n+15y} obtained from identical TCRαβ clonotypes for CMV/pp65 **(C)** or EBV/BMFL1 **(D)** specificity. Coefficient R^2 and p-values from simple linear regression analyses are indicated.

Supplementary Figure 3. Monomeric TCR-pMHC dissociation rates of EBV/BMFL1-specific CD8 TCRαβ clonotypes per healthy donor. (A and B) TCR-pMHC dissociation rates (k_{off}) by wild-type NTAmers (NTA) or mutated NTAmers (CD8null NTA) on a representative selection of EBV/BMFL1-specific T cell clones of each identified TCRαβ clonotype classified according to their color-coded TRBV family from BCL4 and BCL7 **(A)** and BCL2 and BCL9 **(B)**. Unique clonotypes are defined as "others" and depicted in grey. CD8null NTAmer non-binder clones are represented in the grey boxes. (**C**) TCR-pMHC dissociation rates (k_{off}) by wild-type NTAmers (NTA) of a representative selection of EBV/BMFL1-specific T cell clones of each TCRαβ clonotype classified according to their preferential TRAV family usage. Data are depicted as box (25th and 75th percentiles) and whisker (min to max) plots with the middle line indicating the median. Kruskal-Wallis test-derived ($\alpha = 0.05$) p-values are indicated with $^{*}p < 0.05$.

Supplementary Figure 4. Overall TCR-pMHC dissociation rates on bulk virus-specific CD8 T cell populations. (A) Correlations of CMV/pp65-specific TRBV-family frequencies between short-term *ex vivo* expanded bulk CD8 T cell populations and direct *ex vivo* TRBV-stained CD8 T cells (left panel) or *in vitro* single cell cloning (right panel) from BCL4, BCL6, BCL1 and BCL9 at T_n and T_{n+15y}. Coefficient R^2 and p-values from simple linear regression analyses are indicated. **(B)** Correlation of EBV/BMFL1-specific TRBV-family frequencies between short-term *ex vivo* expanded bulk CD8 T cell populations and *in vitro* single cell cloning from BCL7. Coefficient R^2 and p-values from simple linear regression analyses are indicated. (**C**) Overall repertoire TCR-pMHC binding avidity of each donor by *in silico* pooling of k_{off} data for most identified EBV/BMFL1-specific CD8 TCRαβ clonotypes according to their prevalence at T_n and T_{n+15y}. Data are depicted as box (25th and 75th percentiles) and whisker (min to max) plots with the middle line indicating the median. Mann-Whitney (two-tailed) test was performed; ns = not significant.

Supplementary Figure 5: Gene expression and phenotype analyses of CMV/pp65-specific CD8 TCRαβ clonotypes. (A) Volcano plots showing differentially expressed genes between T_n and T_{n+15y} (left panel) and between high and low TCR binding-avidity (right panel) CMV/pp65-specific CD8 T cell clonotypes. Each red dot represents an individual gene with a False Discovery Rate (FDR) < 0.1 (horizontal dotted line). **(B)** Nanostring-based gene

expression on two representative CMV/pp65-specific TCRαβ clonotypes from BCL4, i.e. a high avidity clonotype (clono 1, cl.1H) versus a low avidity clonotype (clono 3, cl.3L), at resting (left panel) and upon CMV/pp65-specific stimulation (right panel). Data are presented as gene expression ratio of clono 1/clono3. **(C)** Percentage of *ex vivo* LILRB1 (left panel) or CD57 (right panel) positive CMV/pp65-specific CD8 T cells found within the following differentiation subsets; effector-memory CD28+ (EM28pos, CCR7-CD45RA-CD28+), effector-memory CD28- (EM28neg, CCR7-CD45RA-CD28-) and terminally differentiated effector (EMRA, CCR7-CD45RA+CD28-). The determination of low (in black) and high (in red) binding avidity clonotypes is based on the known TRBV family usage from BCL4 and BCL6 (n = 10 from 7 independent experiments). **(D)** Representative histograms showing *ex vivo* LILRB1 expression in EBV/BMFL1-specific CD8 T cells according to their respective TRBV family usage.

Supplementary Figure 6. Blocking LILRB1 binding on *ex vivo* cell proliferation of CMV/pp65-specific CD8 TCRαβ clonotypes. CFSE-labeled pp65-specific CD8 T cells of high (clono 1 and clono 2) versus low (clono 3 and clono 4) avidity from BCL4 after treatment with anti-LILRB1 (red line) or an isotype control antibody (gray histogram) and A2/pp65$_{495-503}$ peptide-specific stimulation. Overlays of CFSE fluorescence histograms obtained following combined multimer/pp65- and TRBV-clonotype-specific staining on *ex vivo* treated CD8 T cells at day 4 after stimulation. CFSE-based analysis (% of divided cells, expansion index and replication index) of each CMV/pp65-specific T cell clonotype is shown.

Supplementary Figure 7. Combined LILRB1 and TRBV co-staining to monomeric NTAmer-based off-rate analysis on bulk CMV/pp65-specific CD8 T cell populations. (A) Representative dot plots depicting LILRB1 and TRBV6-5 or TRBV12 co-staining of short-term *ex vivo* expanded bulk CMV/pp65-specific CD8 T cell populations at T_n, T_{n+4y} and T_{n+15y}. **(B)** Compiled TCR-pMHC dissociation rates of TRBV6.5-positive (top panel) or TRBV12-postive (bottom panel) CMV/pp65-specific CD8 T cells at T_n, T_{n+4y} and T_{n+15y} (n = 6 from 2 independent experiments). **(C)** Data are representative of pooled dissociation off-rates (k_{off}, top panel) or ($t_{1/2}$, bottom panel) from 2 independent experiments. The mean value is indicated. p-values by Mann-Whitney test (two-tailed) with $^{**}p < 0.01$.

REFERENCES

1. Martin M. Cutadapt removes adapter sequences from high-throughput sequencing reads. *2011.* 2011;17(1):3.
2. Davis MP, van Dongen S, Abreu-Goodger C, Bartonicek N, and Enright AJ. Kraken: a set of tools for quality control and analysis of high-throughput sequence data. *Methods.* 2013;63(1):41-9.
3. Dobin A, Davis CA, Schlesinger F, Drenkow J, Zaleski C, Jha S, et al. STAR: ultrafast universal RNA-seq aligner. *Bioinformatics.* 2013;29(1):15-21.
4. Anders S, Pyl PT, and Huber W. HTSeq--a Python framework to work with high-throughput sequencing data. *Bioinformatics.* 2015;31(2):166-9.
5. Wang L, Wang S, and Li W. RSeQC: quality control of RNA-seq experiments. *Bioinformatics.* 2012;28(16):2184-5.
6. Love MI, Huber W, and Anders S. Moderated estimation of fold change and dispersion for RNA-seq data with DESeq2. *Genome Biol.* 2014;15(12):550.
