# Supplementary figures and images for: Overall avidity declines in TCR repertoires during latent CMV but not EBV infection
Source: Front Immunol. 2023 Nov 20;14:1293090. doi: 10.3389/fimmu.2023.1293090 (PMC10694213; doi:10.3389/fimmu.2023.1293090)

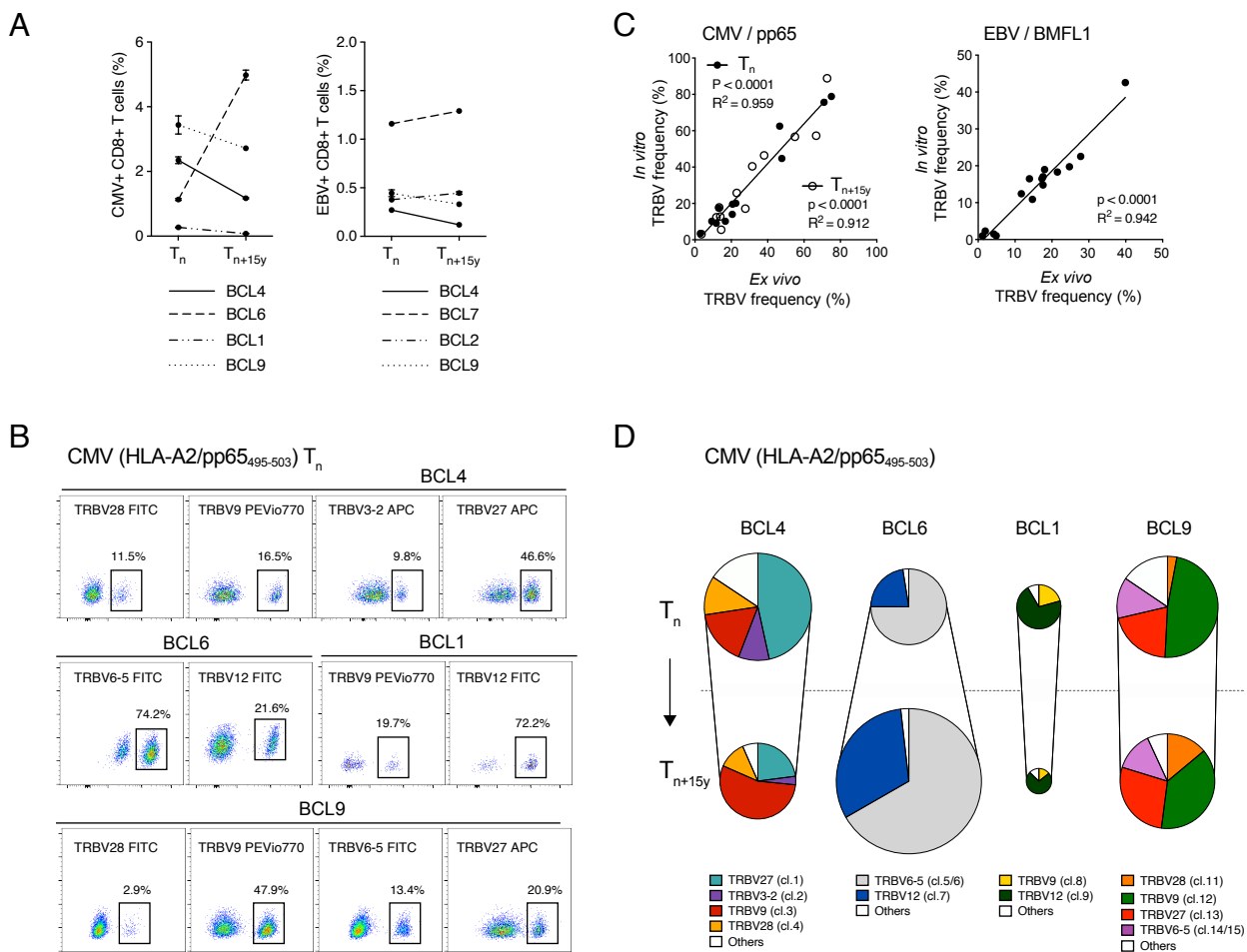

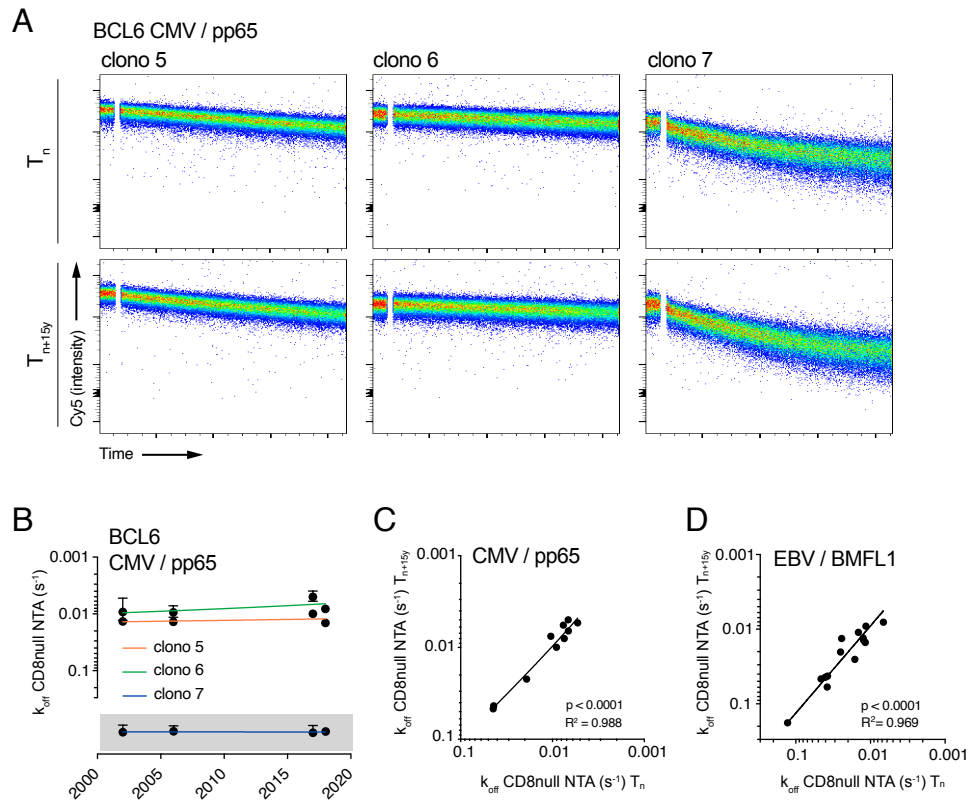

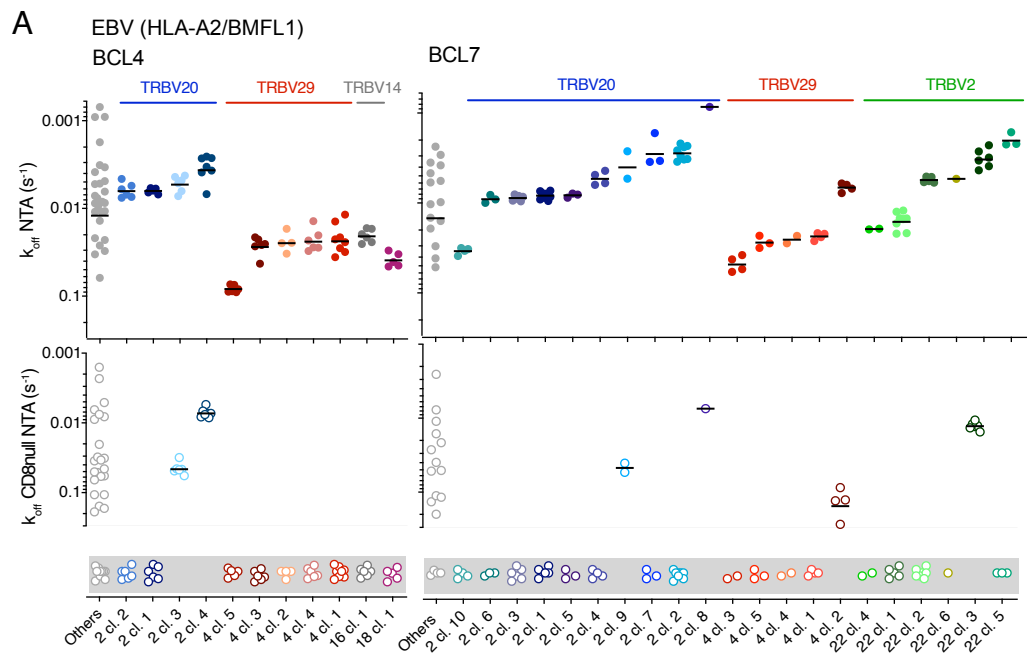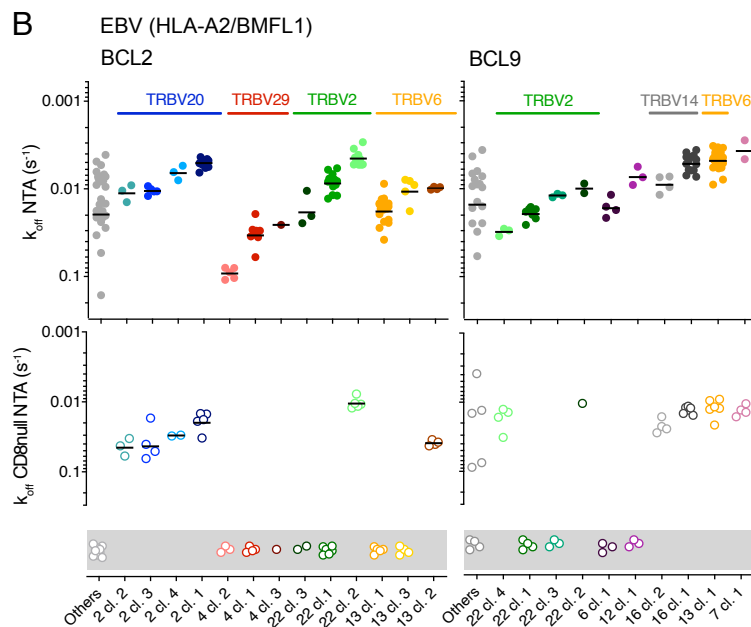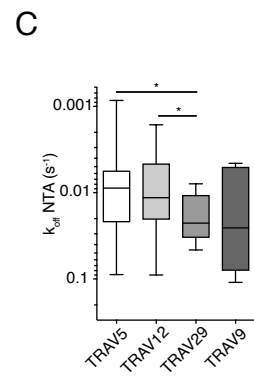

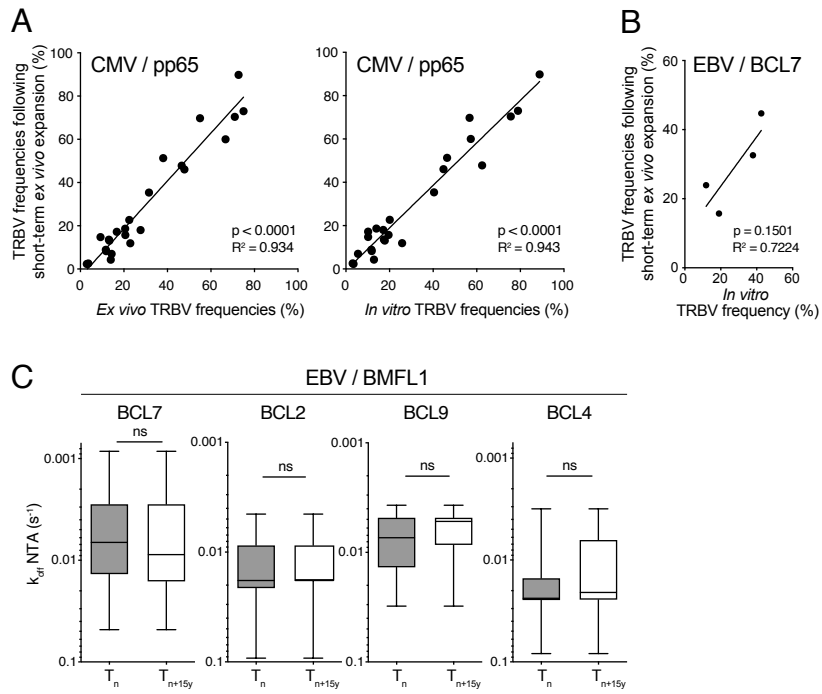

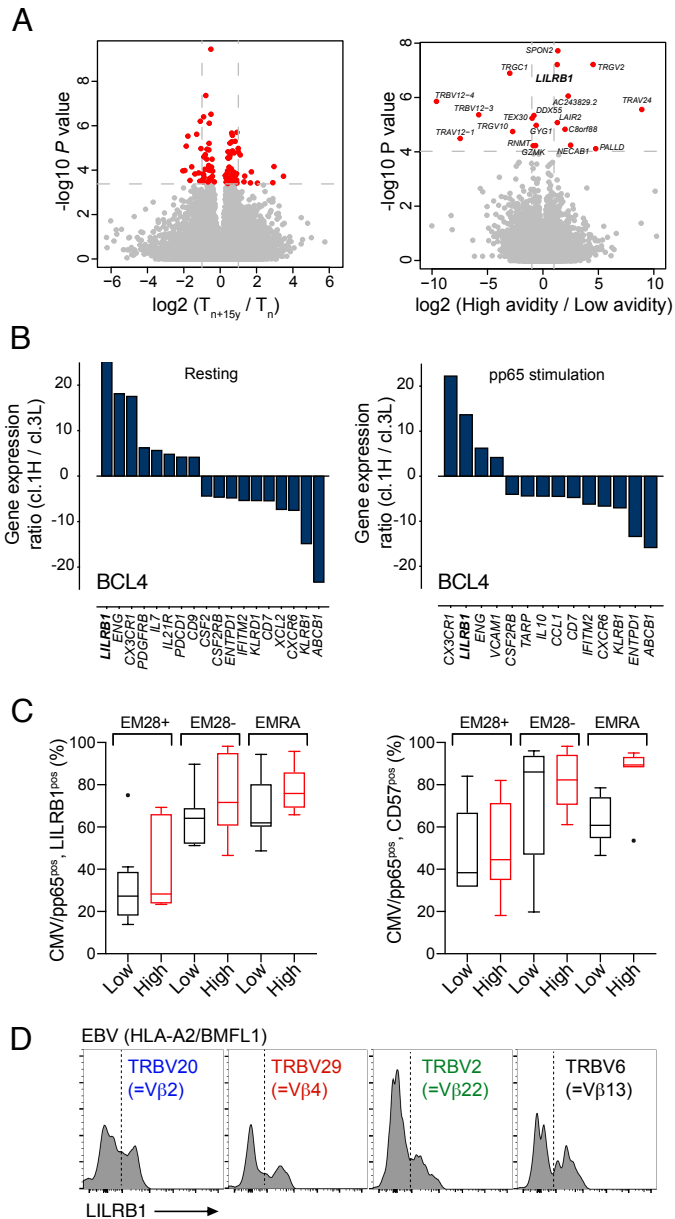

# CMV / BCL4

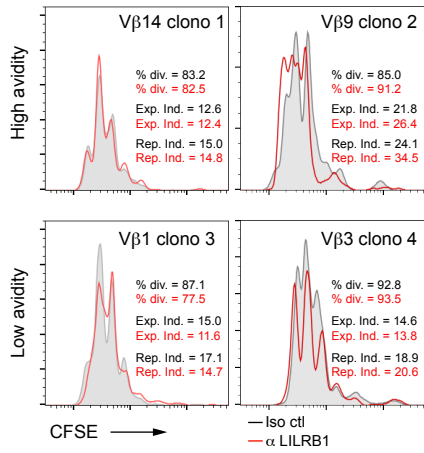

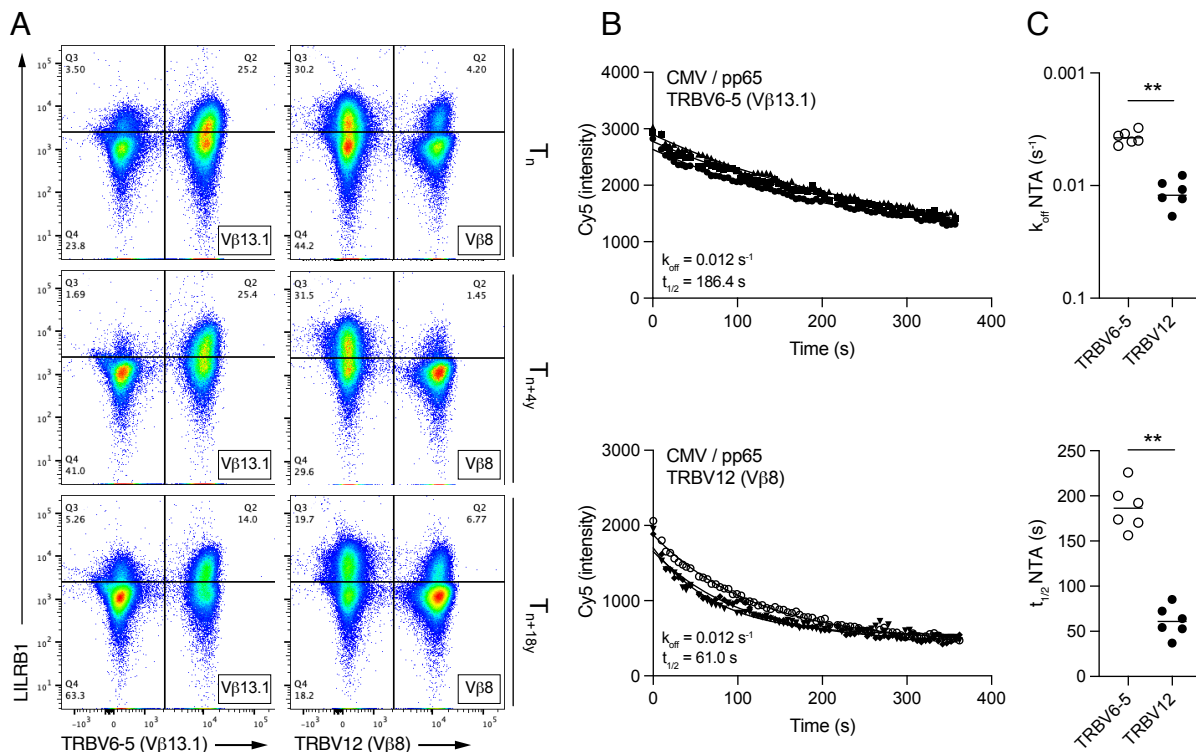

Supplement: Supplementary file 2 [file DataSheet_2.pdf]
